# Association between age of cannabis initiation and gray matter covariance networks in recent onset psychosis

**DOI:** 10.1038/s41386-021-00977-9

**Published:** 2021-03-03

**Authors:** Nora Penzel, Linda A. Antonucci, Linda T. Betz, Rachele Sanfelici, Johanna Weiske, Oliver Pogarell, Paul Cumming, Boris B. Quednow, Oliver Howes, Peter Falkai, Rachel Upthegrove, Alessandro Bertolino, Stefan Borgwardt, Paolo Brambilla, Rebekka Lencer, Eva Meisenzahl, Marlene Rosen, Theresa Haidl, Lana Kambeitz-Ilankovic, Stephan Ruhrmann, Raimo R. K. Salokangas, Christos Pantelis, Stephen J. Wood, Nikolaos Koutsouleris, Joseph Kambeitz, the PRONIA Consortium, Nikolaos Koutsouleris, Lana Kambeitz-Ilankovic, Mark Sen Dong, Anne Erkens, Eva Gussmann, Shalaila Haas, Alkomiet Hasan, Claudius Hoff, Ifrah Khanyaree, Aylin Melo, Susanna Muckenhuber-Sternbauer, Janis Kohler, Omer Faruk Ozturk, David Popovic, Adrian Rangnick, Sebastian von Saldern, Rachele Sanfelici, Moritz Spangemacher, Ana Tupac, Maria Fernanda Urquijo, Johanna Weiske, Antonia Wosgien, Joseph Kambeitz, Stephan Ruhrmann, Marlene Rosen, Linda Betz, Theresa Haidl, Karsten Blume, Mauro Seves, Nathalie Kaiser, Nora Penzel, Tanja Pilgram, Thorsten Lichtenstein, Julian Wenzel, Christiane Woopen, Stefan Borgwardt, Christina Andreou, Laura Egloff, Fabienne Harrisberger, Claudia Lenz, Letizia Leanza, Amatya Mackintosh, Renata Smieskova, Erich Studerus, Anna Walter, Sonja Widmayer, Rachel Upthegrove, Stephen J. Wood, Katharine Chisholm, Chris Day, Sian Lowri Griffiths, Mariam Iqbal, Mirabel Pelton, Pavan Mallikarjun, Alexandra Stainton, Ashleigh Lin, Raimo K. R. Salokangas, Alexander Denissoff, Anu Ellila, Tiina From, Markus Heinimaa, Tuula Ilonen, Paivi Jalo, Heikki Laurikainen, Maarit Lehtinen, Antti Luutonen, Akseli Makela, Janina Paju, Henri Pesonen, Reetta-Liina Armio (Saila), Elina Sormunen, Anna Toivonen, Otto Turtonen, Ana Beatriz Solana, Manuela Abraham, Nicolas Hehn, Timo Schirmer, Paolo Brambilla, Carlo Altamura, Marika Belleri, Francesca Bottinelli, Adele Ferro, Marta Re, Emiliano Monzani, Mauro Percudani, Maurizio Sberna, Armando D’Agostino, Lorenzo Del Fabro, Giampaolo Perna, Maria Nobile, Alessandra Alciati, Matteo Balestrieri, Carolina Bonivento, Giuseppe Cabras, Franco Fabbro, Marco Garzitto, Sara Piccin, Alessandro Bertolino, Giuseppe Blasi, Linda A. Antonucci, Giulio Pergola, Grazia Caforio, Leonardo Faio, Tiziana Quarto, Barbara Gelao, Raffaella Romano, Ileana Andriola, Andrea Falsetti, Marina Barone, Roberta Passatiore, Marina Sangiuliano, Rebekka Lencer, Marian Surman, Olga Bienek, Georg Romer, Udo Dannlowski, Eva Meisenzahl, Frauke Schultze-Lutter, Christian Schmidt-Kraepelin, Susanne Neufang, Alexandra Korda, Henrik Rohner

**Affiliations:** 1 Department of Psychiatry and Psychotherapy, Faculty of Medicine and University Hospital of Cologne, University of Cologne, Cologne, Germany; 2 Department of Psychiatry and Psychotherapy, Ludwig-Maximilian-University, Munich, Germany; 3 Department of Education, Psychology, Communication, University of Bari, Bari, Italy; 4 Max-Planck School of Cognition, Leipzig, Germany; 5 Institute of Nuclear Medicine, Inselspital, Bern University, Bern, Switzerland; 6 School of Psychology and Counselling, Queensland University of Technology, Brisbane, QLD Australia; 7 Experimental and Clinical Pharmacopsychology, Department of Psychiatry, Psychotherapy, and Psychosomatics, Psychiatric Hospital of the University of Zurich, Zurich, Switzerland; 8 Department of Psychosis Studies, Institute of Psychiatry, Psychology & Neuroscience, King’s College London, London, UK; 9 MRC London Institute of Medical Sciences, Hammersmith Hospital, London, UK; 10 Institute of Clinical Sciences, Faculty of Medicine, Imperial College London, London, UK; 11 South London and Maudsley NHS Foundation Trust, London, UK; 12 Institute of Mental Health, University of Birmingham, Birmingham, UK; 13 Department of Neurological and Psychiatric Sciences, University of Bari, Bari, Italy; 14 Department of Psychiatry (UPK), University of Basel, Basel, Switzerland; 15 Department of Psychiatry and Psychotherapy, University of Lübeck, Lübeck, Germany; 16 Department of Neurosciences and Mental Health, Fondazione IRCCS Ca’ Granda Ospedale Maggiore Policlinico, University of Milan, Milan, Italy; 17 Department of Pathophysiology and Transplantation, University of Milan, Milan, Italy; 18 Department of Psychiatry and Psychotherapy, University of Münster, Münster, Germany; 19 Otto Creutzfeldt Center for Behavioral and Cognitive Neuroscience, University of Münster, Münster, Germany; 20 Department of Psychiatry and Psychotherapy, Medical Faculty, Heinrich-Heine University, Düsseldorf, Germany; 21 Department of Psychiatry, University of Turku, Turku, Finland; 22 Melbourne Neuropsychiatry Centre, Melbourne Health, University of Melbourne, Melbourne, VIC Australia; 23 Orygen, Melbourne, VIC Australia; 24 Centre for Youth Mental Health, University of Melbourne, Melbourne, VIC Australia; 25 Max-Planck Institute of Psychiatry, Munich, Germany; 26 University of Cologne, Köln, North Rhineland–Westphalia Germany; 27 Psychiatric University Hospital, University of Basel, Basel, Switzerland; 28 Institute for Mental Health, University of Birmingham, Birmingham, UK; 29 General Electric Global Research Inc, Munich, Germany; 30 University of Milan, Milan, Italy; 31 Department of Neuroscience and Mental Health, Fondazione IRCCS Ca’ Granda Ospedale Maggiore Policlinico, University of Milan, Milan, Italy; 32 Programma 2000, Niguarda Hospital, Milan, Italy; 33 San Paolo Hospital, Milan, Italy; 34 Villa San Benedetto Menni, Albese con Cassano, Italy; 35 Department of Medical Area, University of Udine, Udine, Italy; 36 IRCCS Scientific Institute “E. Medea”, Polo FVG, Udine, Italy; 37 University of Bari Aldo Moro, Bari, Italy; 38 Department of Psychiatry and Psychotherapy, Westfaelische Wilhelms-University Muenster, Muenster, Germany; 39 Department of Psychiatry and Psychotherapy, University Düsseldorf, Düsseldorf, Germany

**Keywords:** Psychosis, Neuroscience, Risk factors

## Abstract

Cannabis use during adolescence is associated with an increased risk of developing psychosis. According to a current hypothesis, this results from detrimental effects of early cannabis use on brain maturation during this vulnerable period. However, studies investigating the interaction between early cannabis use and brain structural alterations hitherto reported inconclusive findings. We investigated effects of age of cannabis initiation on psychosis using data from the multicentric Personalized Prognostic Tools for Early Psychosis Management (PRONIA) and the Cannabis Induced Psychosis (CIP) studies, yielding a total sample of 102 clinically-relevant cannabis users with recent onset psychosis. GM covariance underlies shared maturational processes. Therefore, we performed source-based morphometry analysis with spatial constraints on structural brain networks showing significant alterations in schizophrenia in a previous multisite study, thus testing associations of these networks with the age of cannabis initiation and with confounding factors. Earlier cannabis initiation was associated with more severe positive symptoms in our cohort. Greater gray matter volume (GMV) in the previously identified cerebellar schizophrenia-related network had a significant association with early cannabis use, independent of several possibly confounding factors. Moreover, GMV in the cerebellar network was associated with lower volume in another network previously associated with schizophrenia, comprising the insula, superior temporal, and inferior frontal gyrus. These findings are in line with previous investigations in healthy cannabis users, and suggest that early initiation of cannabis perturbs the developmental trajectory of certain structural brain networks in a manner imparting risk for psychosis later in life.

## Introduction

Schizophrenia (SZ) is viewed as a neurodevelopmental disorder wherein disruptions in the typical trajectory of brain development interact with environmental factors to precipitate psychosis [1]. In this scenario, adolescence is an important time window for the early identification of risk and timely intervention [2], as the adolescent brain undergoes ongoing maturational processes, which include synaptic pruning [3] and maturation of neurotransmitter systems, including the endogenous cannabinoid system [3]. Exposure to environmental stressors during this critical maturation stage might interfere with the normal developmental trajectory of gray and white matter (GM, WM), thereby increasing the risk for developing SZ [1, 4]. One of the most important environmental risk factors for SZ is heavy cannabis use [5, 6]. Given recent international changes in the legality of cannabis use, the investigation of possible harmful effects of the substance on risk groups assumes a new relevance [7]. Cannabis use is associated with structural GM changes in brain regions consistently associated with psychosis [8, 9], including the hippocampus, amygdala, as well as striatal, prefrontal cortical, and cerebellar regions [5, 10]. These GM alterations can be discerned in cannabis-using patients with psychosis [11], prodromal individuals [12] and in healthy individuals who use cannabis regularly [13].

Previous studies indicate that the effect of cannabis on brain structure might be moderated by the age at initiation of heavy use [14]. Healthy cannabis users who had begun to consume cannabis before 16–17 years of age show GM volume (GMV) reductions in the frontal lobe and the parahippocampal gyrus [5, 14, 15] and GMV increases in the cerebellum [13].

Despite this background, other studies of cannabis use in adolescents [16, 17] and retrospective studies investigating the structural-anatomic effects of age of cannabis initiation in adults [18] do not report alterations in GMV. Thus, results focusing on the impact of age of cannabis initiation on brain structure remain inconclusive and focus on the effects of age of initiation in healthy individuals, thereby not considering possible specific effects of the age of cannabis initiation on the earlier trajectory of brain development in psychotic individuals [19]. To date, such studies in psychosis have only investigated the general impact of cannabis use on brain structure. Previous investigations have used univariate approaches, such as region-of-interest analysis or voxel-based morphometry (VBM) [20], thereby neglecting from consideration the highly interconnected nature of the brain [21, 22]. Especially in terms of brain maturation, this interconnectivity plays an important role, since the covariance between brain voxels is thought to reflect shared maturational processes and functional specialization, which might be disrupted in parallel in the face of environmental stressors [23–25]. Multivariate, data-driven approaches such as source-based morphometry (SBM) represent a well-established alternative approach that accommodates the covariance between brain voxels [26, 27]. In SBM, an independent component analysis (ICA) identifies brain networks characterized by covariation in GMV [26, 27]. The approach thus enables the comparison of independent structural brain networks between different groups [26, 27]. By maximizing the independence of isolated brain networks, SBM is a powerful technique for separating scanner noise (e.g., often reported site-effects) from true signals [28], and is thought to unify structural regions that have comparable maturational trajectories. The recently introduced semi-blind ICA algorithms, such as group information guided ICA (GIG-ICA), incorporate prior information in the form of spatial constraints [29, 30], thus exploiting the advantages of data-driven approaches, while focusing the analysis on networks of interest [29].

In the current study, we aimed to investigate the effect of the age of cannabis initiation among patients with recent onset psychosis (ROP) on structural networks that are already reliably associated with SZ and thus are of relevance for the pathology of the disease. Due to the compilation of data from multiple sites, we concentrated our analyses on networks that are robustly associated with alterations in patients with SZ across sites. A recent study by Gupta et al. [9] merged data from nine different studies and identified four structural components of abnormal GMV covariation with high reproducibility. We hypothesized that the age of cannabis use initiation in ROP patients is associated with alterations in SZ-related GM networks, including brain regions previously associated with early initiation of cannabis use in healthy individuals (e.g., frontal areas and cerebellum). We aimed to advance the present knowledge of cannabis effects on the four components reported for SZ patients: (i) superior temporal gyrus, inferior frontal gyrus and insula, (ii) superior frontal gyrus, middle frontal, and medial frontal gyrus, (iii) brainstem, and (iv) inferior semilunar lobule and cerebellar tonsils. Previous work indicated that GM concentration was reduced in the frontal, temporal, and cerebellar components (i, ii, iv) and increased in the brainstem component, (iii) in SZ patients compared to controls [9]. Further, we adopt a network-based perspective to explore the associations of the age of cannabis initiation on the psycho- and neuropathology of psychosis. We predicted that this approach might reveal potential pathways whereby cannabis use patterns might propagate to positive psychotic symptoms and/or development of neurostructural perturbations [31].

## Materials and methods

### Study design and population

We analyzed data of 102 patients with ROP aged 15–40 years from two studies, the multisite longitudinal PRONIA study (www.pronia.eu, German Clinical Trials Register identifier DRKS00005042 [32]) and the ongoing, monocentric, longitudinal Cannabis Induced Psychosis (CIP) study, after harmonizing the study protocols (Supplementary Fig. 1). CIP patients were recruited at the Department of Psychiatry at the Ludwig Maximilian’s University of Munich, while ROP cases included in PRONIA were recruited at eight European sites (see [32]). ROP experienced an affective or non-affective psychotic episode within the past 24 months and present within the 3 months preceding study entry. Psychiatric diagnoses were obtained by trained clinical raters, based on the Structured Clinical Interview for DSM-IV disorders [33]. To focus our analysis on ROP patients who had a clinically-relevant comorbid cannabis use, we imposed additional inclusion criteria, defined by (i) cannabis use preceding the onset of psychotic symptoms by no more than 2 weeks as defined in the International Classification of Diseases, 10th Revision, criteria for substance-induced psychosis [34], and/or (ii) a lifetime cannabis abuse or dependence [33]. Participants were only included when their age of cannabis initiation was recorded (Supplementary Information for detailed inclusion and exclusion criteria, Supplementary Fig. 5). Fourteen subjects from the PRONIA study had to be excluded due to the lack of this information. Subjects with missing data for age of cannabis initiation had significantly more severe symptoms, were more likely to have cannabis abuse or dependency use, and there was an interaction with site (Supplementary Table 3).

All individuals from PRONIA underwent baseline assessment between 2014 and 2019 and were followed for up to 36 months. The CIP recruitment took place from December 2016 until May 2019 and the follow-up period was 9 months. Most assessments overlapped between the two studies in that both studies included multimodal imaging, a neuropsychological assessment and a clinical protocol (assessments are listed in Supplementary Table 1).

Prior to their inclusion in the study, all participants provided written informed consent (either personally or through a legal guardian if below the age of 18). Studies were approved at their respective sites by the local research ethics committees.

### Assessment of cannabis consumption

The age of initiation and other cannabis intake measures were assessed in a clinical interview. Initiation age entered all models as a continuous variable (Supplementary Fig. 3 for distribution of age of cannabis initiation). For the purpose of tabular presentation, we divided the study sample into early- and late-onset users, based on the median of 17 years (Supplementary Table 4 for age of cannabis initiation as continuous variable) [15].

### Acquisition protocol and preprocessing pipeline of structural MRI

A harmonized protocol for the acquisition of structural MRI data was used at all sites. For preprocessing, we used the open-source CAT12 toolbox (version r1155; http://dbm.neuro.uni-jena.de/cat12/), which is an extension of SPM12 running in MATLAB 2018a. First, all images were segmented into GM, WM, and cerebrospinal fluid, normalized to stereotactic space of Montreal Neurological Institute (MNI-152) and multiplied with the Jacobian determinants obtained during registration to derive the final GMV maps. After quality control (Supplementary Information), we regressed age and sex effects voxel-wise, as previous studies have shown that regressing for such effects prior to SBM analysis increases sensitivity to group differences [28]. Subsequently, images were realigned to a two mm voxel resolution and smoothed with a ten mm (full-width at half maximum) Gaussian kernel [9] (Supplementary Table 5).

### Source-based morphometry

**Fig. 1:**
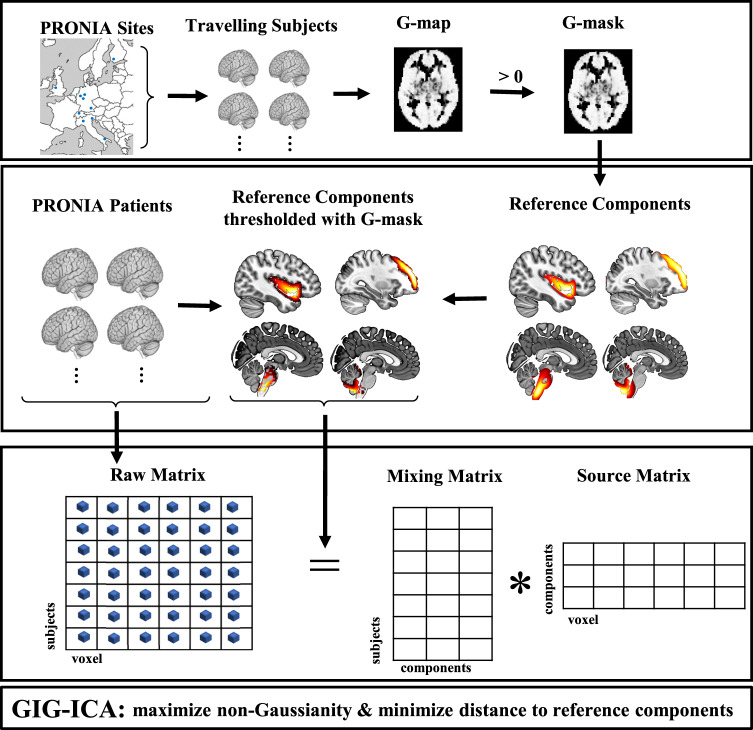
For the analysis pipeline based on GIG-ICA four components from a previous study of SCZ [9] were selected as reference components and thresholded using the G-theory mask (derived from six healthy traveling subjects) to correct for scanner-specific effects. We extracted components using GIG-ICA by maximizing the non-Gaussianity and simultaneously minimizing the distance to the reference components.

SBM analysis was conducted by applying GIG-ICA to sMRI data using the GIFT toolbox (http://mialab.mrn.org/software/gift/) in MATLAB 2020. Here, GIG-ICA was optimized to identify four independent components with maximum similarity to the four reference components (RCs) that had previously been associated with SZ, with high reproducibility between sites [9]. To account for study-specific scanner effects, we used a strategy based on G-theory for voxel selection of the RCs [32], employing a threshold of >0 to exclude voxels showing only between-site but no inter-subject-variation [35] (Fig. 1, Supplementary Information).

In the first step, GM images were converted to one-dimensional row vectors and concatenated across participants. After excluding outliers with extremely high source variability that might otherwise drive spurious significant results (Supplementary Information), we derived a 102-by-175,000 GMV voxel matrix. This matrix was decomposed into a source matrix and a mixing matrix. The mixing matrix represents loadings, i.e., the weights of individual participants on each component. The source matrix, represents the relationship between each voxel and the components (Fig. 1). This decomposition simultaneously maximizes the correspondence to the RCs and the independence of the components from each other such that each row in the source matrix is maximally independent from the others. To match the components derived from this study with the established RCs and to test their validity, we used a stepwise procedure as described previously [36] (Supplementary Information). Based on that previous literature [36], components from the current study (COIs) with a correlation *r* > 0.5 with a RC were included in subsequent analyses. Additionally, we utilized GIG-ICA on a subsample restricted to cases of schizophrenia spectrum disorder (SSD) to enhance comparability with the sample in which the RCs were generated and to further reduce heterogeneity in terms of severity of symptoms and diagnoses. To test for sex-specific effects of cannabis use initiation on brain structure we recalculated the components based on male subjects only. Due to the lower sample size, this analysis was not possible in females.

#### Voxel-based morphometry

To maximize comparability with previous studies, we performed an additional VBM analysis (Supplementary Information).

### Statistical analysis

We performed all further statistical analyses using R language for statistical computing, version 3.6.2 [37]. To analyse differences in GMV covariation due to age of cannabis initiation, we investigated participants’ loading coefficients, employing a linear mixed effects model in the R-package “lmerTest” [38] with loading coefficients as dependent variables. Age of cannabis initiation was modeled as a fixed effect, while the factor “site” was modeled as a random effect. In our analysis of ICA components, larger loading coefficients indicate a stronger weighting of the spatial pattern in the individuals [9, 26]. Higher loading coefficients, coupled with a positive spatial component, shall be interpreted as greater GMV in this component [9]. Effect sizes in terms of *R*^*2*^ were calculated with the R-package “r2glmm” as proposed recently [39]. To assess the possible impact of certain confounding factors typically reported to effect GMV, we estimated the same model with addition of the duration of heaviest use, the chlorpromazine equivalent cumulative in lifetime, current medication use (yes/no) [40, 41], alcohol abuse (yes/no) [42] and the duration of illness [43] (all variables modeled as fixed factors). All *p* values were corrected for multiple testing using the false discovery rate (FDR) with a threshold of *p*_FDR_ < 0.05 [44]. Associations between age of cannabis use initiation and COI loadings were tested independently with cognition (Supplementary Information).

### Exploratory network analysis

We fitted a network in the form of a Gaussian Graphical Model (using the R-package “qraph”, version 1.6. 3 [45]), including all COI loadings passing the inclusion threshold, initiation age, duration of use, and positive psychotic symptoms in the last 7 days measured by the Positive and Negative Syndrome scale (PANSS) [46]. In such a network, undirected connections between two variables represent pairwise partial correlations after conditioning on all other variables [47]. These connections are interpreted as predictive effects between two variables, which cannot be explained by any other variable in the network. We determined the optimal network model by using stepwise, unregularized model selection and tested robustness and stability (Supplementary Information).

## Results

### Study population

ROP patients with early (<17 years) and late (≥17 years) cannabis use initiation showed no differences in sociodemographic variables (Table 1 and Supplementary Table 2). Early users had a significantly longer mean duration of cannabis use compared to late users, had more severe positive psychotic symptoms and significantly longer duration between the cannabis use initiation and first attenuated psychotic symptoms measured by Standardized Interview for the assessment of Prodromal Symptoms (modified version 5.0) (any SIPS-P item ≥3) [48]. Patients with early- and late-initiation of cannabis did not differ for cumulative frequency of cannabis use in the previous 3 months, prevalence of a SCID lifetime diagnosis of cannabis abuse or dependency, prevalence of lifetime alcohol abuse, other drugs taken or in the cumulative dose or current intake of antipsychotics.

**Table 1:** Demographics and clinical data.

	Early (<17)	Late (17+)	df	*T*/*Z*/*X*^2^	*p* value
*Samples and study variables*					
Sample sizes	58	44			
CIP (%)	29 (49.2)	15 (34.1)	1	1.970	0.160
Age [mean (SD) years]	24.1 (4.1)	23.4 (4.0)	94	0.820	0.413
Sex [F (%)]	11 (19.0)	12 (27.3)	1	0.571	0.450
*Sample size per site*			7	22.690	0.002
Munich (%)	45 (77.6)	25 (56.8)			
Milan Niguarda (%)	0 (0)	6 (13.6)			
Basel (%)	8 (13.8)	3 (6.8)			
Cologne (%)	2 (3.4)	3 (6.8)			
Birmingham (%)	3 (5.2)	0 (0)			
Turku (%)	0 (0)	5 (11.4)			
Udine (%)	0 (0)	1 (2.3)			
Düsseldorf (%)	0 (0)	1 (2.3)			
*Cannabis use*					
Lifetime history of DSM-IV cannabis use disorder [*N* (%)]			2	1.030	0.597
Cannabis abuse (%)	24 (41.4)	23 (22.5)			
Cannabis dependency (%)	25 (24.5)	16 (15.7)			
Initiation age [mean (SD) years]	14.9 (1.2)	19.8 (3.2)	51.400	−9.61	<0.001
Cumulative months lifetime [mean (SD) months]	58.1 (40. 5)	25.2 (24.9)	55.990	3.831	<0.001
Duration of heaviest use [mean (SD) days]	822.2 (873.6)	389.7 (379.1)	70.637	3.197	0.006
*Level of use in the heaviest use period (%)*			2	2.889	0.236
>10 times per month/dependency	49 (48.0)	35 (34.3)			
<10 times per month	5 (5.9)	6 (5.9)			
Only once	3 (2.9)	0 (0)			
Duration since last use [mean (SD) days]	369.1 (1151.8)	276.6 (661.6)	87.127	0.489	0.626
*Level of use in the last 3 months—cumulative frequency (%)*			7	3.106	0.875
0 times	16 (15.7)	16 (15.7)			
1–5 times	3 (2.9)	4 (3.9)			
6–10 times	4 (3.9)	2 (2.0)			
11–15 times	3 (2.9)	2 (2.0)			
16–20 times	2 (2.0)	2 (2.0)			
21–30 times	2 (2.0)	1 (1.0)			
>30 times	14 (13.7)	6 (5.9)			
*Psychopathology [mean (SD)]*					
Positive and negative syndrome scale—positive	20.7 (5.3)	17.8 (6.9)	72.541	2.293	0.025
Positive and negative syndrome scale—negative	14.7 (5.47)	14.4 (5.8)	83.919	1.031	0.306
Positive and negative syndrome scale—general	35.0 (8.3)	33.2 (8.3)	81.319	0.231	0.818
Onset age of psychotic disorder	23.6 (4.2)	23.3 (3.8)	93.776	0.399	0.691
Years between first cannabis use initiation and attenuated psychotic symptoms—years [mean (SD)]	7.5 (4.6)	3.3 (4.0)	66.025	4.175	<0.001
Years between initiation of heaviest cannabis use and attenuated psychotic symptoms—years [mean (SD)]	2.6 (3.9)	1.4 (2.6)	70.393	1.524	0.132
*Medication [mean (SD)]*					
Currently treated (%)	37 (63.8)	28 (63.6)	1	<0.001	1
Chlorpromazine equivalent (cumulative lifetime)	4764.1 (7445.2)	4903.0 (7872.9)	87.852	−0.089	0.929

### Creation of the components of interest (COIs)

**Fig. 2:**
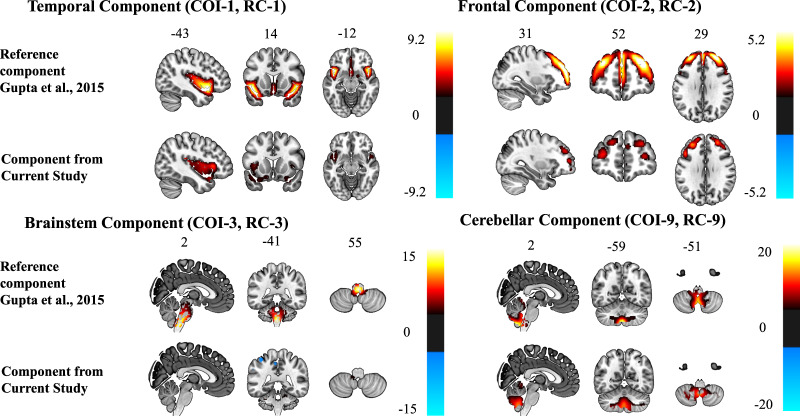
Cerebral mapping of the reference components and the four components from the current study, all thresholded at |z| > 2.5. The reference components are thresholded with the G-theory mask.

SMRI data of 102 ROP patients with history of clinically-relevant cannabis use were decomposed into four components based on independence and correspondence to the RCs [9]. Brain regions of all four COIs were identified from the Talairach Daemon (http://www.talairach.org/daemon.html) and visualized with the MRIcroGL software (McCausland Center for Brain Imaging, University of South Carolina; https://www.nitrc.org/projects/mricrogl/). A minimum z-threshold was set to |>2.5| and a maximum z-threshold was derived from [9] for each component independently (Fig. 2).

Three of the four COIs passed the threshold of *r* > 0.5 for correspondence to the RCs and were included in further analyses, while COI-3, which comprises mainly the brainstem, was excluded (*r* = 0.38, *p* < 0.001).

COI-2 had the highest correlation with RC-2 and encompassed mainly the superior, medial, and middle frontal gyrus (*r* = 0.712, *p* < 0.001, Table 2). The next highest correlation was between COI-1 and RC-1 (*r* = 0.671, *p* < 0.001). COI-1 comprised mainly the superior temporal, precentral, frontal and parahippocampal gyrus and insula (Table 2). COI-9 had a moderate correlation with RC-9 (*r* = 0.59, *p* < 0.001), although many voxels had to be excluded from RC-9 due to study-specific scanner effects (41%). This component mainly comprised cerebellar regions (Table 2). The assignment between COIs and RCs remained similar in our control subanalyses restricted to the male and SSD groups, except that the correlation coefficient was lower (Supplementary Information).

**Table 2:** Statistical measures and brain coordinates of the identified brain region components.

Component	Reference component (Pearson *r*^*2*^*)*	Loadings direction	LME (initiation age)			Brain region label	*L*/*R* Volume in cm^3^	Brodmann area	L/R Max *z*-value (MNI coordinates)
			*t (df)*	*p*_FDR_	*R*^2^/*R*^2^ whole model				
COI-1	Temporal (0.68)	Early > late	−0.474 (96)	0.64	0.002/0.019	Insula	8.4/7.6	13, 40	5.4 (−38, 8, 4)/4.3 (44, −28, 18)
						Inferior frontal gyrus	5.2/3.4	13, 44, 45, 47	5.4 (−40, 22, 2)/3.6 (26, 14, −24)
						Extra nuclear	1.5/0.4	13, 47	5.0 (−36, 20, 0)/3.2 (36, 0, 10)
						Precentral gyrus	0.6/1.0	6, 13, 44	4.4 (−40, 8, 8)/3.5 (48, −6, 6)
						Superior temporal gyrus	7.2/6.6	13, 21, 22, 38, 41	3.9 (−42, 14, −22)/3.9 (30, 14, −26)
						Parahippocampal gyrus	1.0/0.3	28, 34	3.3 (−20, −6, −20)/2.8 (20, −8, −18)
COI-2	Frontal (0.72)	Late > early	0.656 (93)	0.64	0.016/0.047	Superior frontal gyrus	6.3/6.0	6, 8, 9, 10, 11	4.3 (−26, 40, 34)/3.8 (26, 44, 32)
						Middle frontal gyrus	8.0/4.0	8, 9, 10, 46, 47	4.0 (−30, 40, 32)/3.6 (30, 44, 30)
						Medial frontal gyrus	1.1/0.8	9, 10	3.6 (−24, 36, 32)/2.9 (6, 54, 24)
COI-3	Brainstem (0.38)	Early > late	–	–	*–*	*Uncus*	*1.5/1.2*	*20, 28, 36, 38*	*5.9 (−28, 0, −42)/4.0 (26, −6, −36)*
						*Middle temporal gyrus*	*1.5/0.4*	*21*	*5.0 (−32, 8, −42)/3.2 (44, −70, 16)*
						*Superior temporal gyrus*	*1.3/0.1*	*22, 38*	*4.6 (−24, 8, −40)/2.6 (52, 14, −18)*
						*Inferior parietal lobule*	*1.5/0.0*	*40*	*3.8 (−38, −44, 58)/NA (0, 0, 0)*
COI-9	Cerebellum (0.59)	Early > late	−2.758 (100)	0.02	0.079/0.100	Cerebellar tonsil	4.0/3.8	–	12.2 (0, −56, −46)/10.5 (4, −56, −46)
						Inferior semilunar lobule	3.1/1.2	–	11.7 (−4, −58, −50)/10.9 (4, −58, −50)
						Nodule	0.8/1.2	–	8.5 (−4, −56, −38)/9.0 (0, −54, −38)
						Uvula	1.3/1.7	–	7.7 (−4, −64, −42)/7.2 (0, −62, −38)
						Culmen	1.0/1.2	–	4.3 (−2, −50, −6)/4.6 (2, −50, −6)

Bold italic refers to negative component regions.

*LME* linear mixed effects model.

### Relationship between the components and cannabis use patterns

**Fig. 3:**
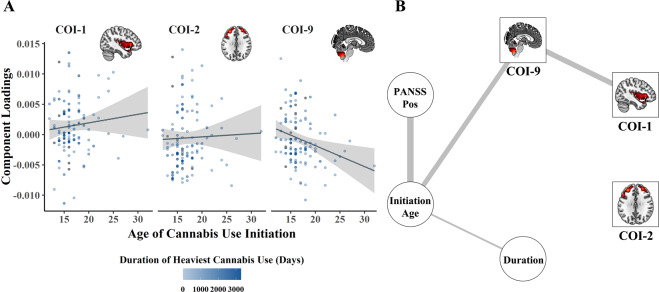
Association between components of interest (COIs), cannabis, and clinical measures. Age of cannabis use initiation, duration of heaviest use and components (**A**). Network of identified components, cannabis measures and PANSS positive scale (**B**). Edges represent partial correlations between the nodes. Each edge is corrected for all other edges in the network and the scaling of edges in width and color saturation were adjusted by setting the cut-argument in qgraph to 0.2 [35]. All correlations in the network are negative.

We found that higher loading coefficients for COI-9 were significantly associated with earlier cannabis initiation, after correcting for site (*t*_102_ = −2.762, *p*_FDR_ = 0.02). In combination with the predominantly positive component, this implies that increased GMV in cerebellar regions is associated with an earlier initiation of cannabis use. Adding several hypothesized confounding covariates had slight effects on the results, significantly higher loading coefficients were still associated with an earlier initiation (*t*_80_ = −3.00, *p*_FDR_ = 0.01), with the difference that the random effect “site” became significant in this model (*t*_80_ = 2.62, *p*_FDR_ = 0.03). No other covariates significantly correlated with the loading coefficients of any of the components. Age of cannabis initiation explained 7.9% of the 10% of the variance explained in the full model. Initiation age did not show any significant effect on the other components (Fig. 3). Cognitive performance was neither associated with the age of cannabis initiation nor with the cerebellar loadings (Supplementary Information). Subsequent control analyses, in the SSD and the male subgroups showed comparable effects (Supplementary Information).

### Complementary network analysis

The network (Fig. 3) illustrates the connections between the overall severity of positive symptoms at time of assessment measured by PANSS, age of cannabis initiation, duration of use, and COI-2, COI-1, and COI-9. From 15 possible edges, only four remained in the unregularized model selection procedure. Age of cannabis initiation was negatively associated with positive symptoms measured by the PANSS, age of initiation was negatively associated with the duration of cannabis use, age of initiation was negatively associated with COI-9, whereas COI-9 was negatively associated with COI-1. These associations are specific, i.e., they remain after all other associations have been taken into account. Bootstrapping analyses showed that edges retained in the final model were also present in the majority of bootstrapped networks (Supplementary Fig. 6). However, when testing case subsetting, the edges were not stable (Supplementary Fig. 7).

#### Voxel-based morphometry

We did not find any significant volume difference associated with the age of cannabis initiation at the proposed threshold of FWE—*p* < 0.05. However, at an uncorrected threshold (*p* < 0.005, *k* = 5) the direction of our VBM analyses was in line with our findings in SBM (Supplementary Table 10 and Supplementary Fig. 8).

## Discussion

To the best of our knowledge, this is the first study investigating the effects of the age of cannabis use initiation on GMV in patients with ROP. Moreover, for the first time, we employed a GIG-ICA to investigate cannabis use history on structural brain networks previously identified in SZ [9]. Our results link the initiation of cannabis use during adolescence to long-lasting structural effects, manifesting in the present finding of greater GMV in patients with an earlier age of cannabis initiation. Notably, this effect was specific for the age of initiation, and was robust to several possible confounding factors often discussed in the literature [14, 49]. Furthermore, we provide evidence that an earlier age of initiation is specifically associated with more severe positive psychotic symptoms in ROP. The presence of greater volume in the cerebellar network was associated with reduced GMV in COI-1, a network mainly comprising the insula, superior temporal, and inferior frontal gyrus, which has previously been shown to best discriminate between HC and SZ [9].

The positive association between greater GMV in the cerebellum and earlier cannabis use initiation is in line with previous structural imaging studies comparing cannabis-using and non-using healthy adolescents [50, 51] and young adults [14, 50–53]. In recent decades the cerebellum has been increasingly associated with higher cognitive functions, such as emotion regulation, working memory, and language [54], all of which undergo substantial evolution during adolescence. Notably, in the current study, cognition was not associated neither with cerebellar GMV, nor with age of cannabis initiation. Previous literature has indicated an impact of cannabis on cognitive performance, however our findings are in line with meta-analytic evidence showing the absence of a mediating effect of age of initiation [55]. Surprisingly, there was no association between the cerebellar network and cognitive performance [54]. These unexpectedly negative findings might be due particulars of our selection of cognitive domains. The cerebellum is amongst the latest brain structures to mature. It has an inverted U-shaped neurodevelopmental trajectory, attaining a GMV peak in adolescence, which then declines in early adulthood [54]. A finding of increased cerebellar volume persisting into adulthood has therefore been interpreted to indicate a disturbance of typical brain maturation, such as failure of synaptic pruning [13, 14, 51]. Remarkably, a twin study of healthy brain development indicates weaker genetic effects on cerebellar GMV development as compared to all other brain structures [56]. Hence, it might be hypothesized that environmental factors play an important role in shaping cerebellar structure. Cumulative evidence suggests that these effects might be sex-specific due to a more protracted and hence more vulnerable cerebellar development in men [54], thus explaining the increased likelihood for cannabis psychosis in male (male:female, 4:1) [57]. However, due to the limited sample size, we cannot exclude that a comparable effect may also be present in females.

While previous studies often neglected the cerebellum or found a cerebellar decrease in SZ [58, 59], a recent meta-analytic study indicates that psychotic patients had greater cerebellar brain volume as compared to HC [8]. Similarly, individuals at clinical high risk for psychosis exhibit greater volume in cerebellar regions compared to HC [32]. Moreover, alterations in these regions contribute to a brain network predictive for poor psychosocial functioning [32]. Hence, we suggest that present findings might indicate an anatomic signature of psychosis either initiated or exacerbated by early cannabis use.

Surprisingly, we did not find associations between GMV of frontal networks (COI-2) and the age of cannabis use initiation. Volumetric decreases in frontal regions and the parahippocampal gyrus have been associated with an earlier initiation of cannabis use in healthy individuals [14, 15], and in cannabis-using adolescents compared to their abstinent peers [18]. The explanation for our negative result for COI-2 might be threefold. First, we specifically test for an effect of age of cannabis *initiation* in ROP patients, in contrast to previous studies potentially detecting general effects of cannabis use in psychosis. Second, specific effects of cannabis use during adolescent brain maturation might differ in vulnerable individuals later presenting with ROP, due to genetic vulnerability or additional early environmental risk factors [60]. Third, the use of univariate statistics in previous studies hindered exploring the highly interconnected nature of the brain. Despite our negative univariate results, our findings in GMV covariation, which is thought to reflect shared maturational processes [23–25], might suggest that this measure is a particularly important marker of neurodevelopmental perturbations.

The exploratory network analysis revealed a pathway in which the cerebellar network bridges the association between the network that comprises the insula, superior temporal and inferior frontal gyrus and age of cannabis initiation. Interestingly, greater GMV in the cerebellar network associated with earlier age of initiation was in turn associated with decreased GMV in the insula, superior temporal, and inferior frontal network (COI-1). This latter network was the most predictive of SZ and includes brain regions consistently implicated in psychosis [8]. Present findings are consistent with a model that cannabis consumption during adolescence causes an excursion from the typical brain maturational process, thereby increasing vulnerability to develop psychosis later in life. However, causal inferences are fraught, since it cannot be excluded from cross-sectional studies that specific GMV patterns may predispose an earlier cannabis consumption [14], although there is some evidence to the contrary [50]. Interestingly, stronger positive symptoms were associated only with an earlier age of initiation, irrespective of GMV in any brain network, or the duration of the heaviest use. This finding adds to previous studies showing that adolescent cannabis use increases the risk of more severe psychotic symptoms [61, 62]. Moreover, our observation that cannabis use precedes the onset of attenuated psychotic symptoms indicates that this effect is directed, as likewise reported elsewhere [63].

We note some limitations of our study. Although we corrected for inter-scanner effects, some results might yet have been influenced by differing MRI machines and protocols. This possibility might be excluded in future studies by balancing between different sites which would also allow for additional statistical power in support of methods to correct for any site-effects [64]. In the network analysis, final edges were included across most bootstrapped networks, but the resultant network structure was unstable under subsetting of cases. This instability could be due to the marginal sample size (*N* = 102) relative to the number of nodes analysed (*N* = 6). Further, network approaches are typically applied for investigating specific symptoms, such as PANSS subscores. Again, our sample size calls for some reduction of variables. All our analyses are cross-sectional, which limits causal inferences. A longitudinal study design could enable the investigation of directionality of the neuroanatomic effects of cannabis use in psychosis, although requiring a logistically difficult study beginning in early adolescence. Follow-up studies might then investigate the possibly mediating and interacting detrimental effects of other risk factors, such as childhood adversity [65] or inattention-hyperactivity symptoms [66]. Importantly, our study lacks a control condition of healthy cannabis users. Future studies might test whether the effects found in the current study are specific to psychotic patients or represent general effects of cannabis on the developing brain.

Our GIG-ICA approach indicates that earlier age of cannabis use initiation among patients with ROP is specifically associated with increased volume in the same cerebellar network previously identified in SZ patients. We cautiously attribute this increase in cerebellar GMV to interference with the trajectory of typical brain maturation. Additionally, we found evidence that earlier initiation of cannabis use is associated with more severe psychotic symptoms in our ROP group. This result calls for more detailed examination of the interaction between early cannabis use, neurodevelopment perturbation, and risk of psychosis. Since the legalization of cannabis products in many countries shall have unpredictable effects on cannabis consumption in adolescents, it becomes a matter of vital interest to establish the contribution of cannabis use to the burden of risk factors for psychosis.

## Funding and disclosure

The CIP study is a DFG-funded project (grant agreement No. KA 4413/1-1). The PRONIA study is a European Collaboration Project funded under the 7th Framework Program under grant agreement no 602152. RU reports grants from Medical Research Council, grants from the National Institute for Health Research, and personal fees from Sunovion, outside the submitted work. NK and RS received honoraria for talks presented at education meetings organized by Otsuka/Lundbeck. CP participated in advisory boards for Janssen-Cilag, AstraZeneca, Lundbeck, and Servier and received honoraria for talks presented at educational meetings organized by AstraZeneca, Janssen-Cilag, Eli Lilly, Pfizer, Lundbeck, and Shire. CP acknowledges support by an Australian National Health & Medical Research Council (NHMRC) Senior Principal Research Fellowship (ID: 1105825), a NHMRC Program Grant (ID: 1150083). All other authors report no biomedical financial interests or potential competing interests. Open Access funding enabled and organized by Projekt DEAL.

## ^Supplementary information^

Supplementary Material

## References

[1] Murray RM, Bhavsar V, Tripoli G, Howes O (2017). 30 Years on: how the neurodevelopmental hypothesis of schizophrenia morphed into the developmental risk factor model of psychosis. Schizophr Bull.

[2] Antonucci LA, Penzel N, Pergola G, Kambeitz-Ilankovic L, Dwyer D, Kambeitz J (2020). Multivariate classification of schizophrenia and its familial risk based on load-dependent attentional control brain functional connectivity. Neuropsychopharmacology.

[3] Hurd YL, Manzoni OJ, Pletnikov MV, Lee FS, Bhattacharyya S, Melis M (2019). Cannabis and the developing brain: insights into its long-lasting effects. J Neurosci.

[4] Casadio P, Fernandes C, Murray RM, Di Forti M (2011). Cannabis use in young people: the risk for schizophrenia. Neurosci Biobehav Rev.

[5] Murray RM, Englund A, Abi-Dargham A, Lewis D, Di Forti M, Davies C (2017). Cannabis-associated psychosis: neural substrate and clinical impact.. Neuropharmacology..

[6] Marconi A, Di Forti M, Lewis CM, Murray RM, Vassos E (2016). Meta-analysis of the association between the level of cannabis use and risk of psychosis. Schizophr Bull.

[7] Archie SR, Cucullo L (2019). Harmful effects of smoking cannabis: a cerebrovascular and neurological perspective. Front Pharmacol.

[8] Brandl F, Avram M, Weise B, Shang J, Simões B, Bertram T (2019). Specific substantial dysconnectivity in schizophrenia: a transdiagnostic multimodal meta-analysis of resting-state functional and structural magnetic resonance imaging studies. Biol Psychiatry.

[9] Gupta CN, Calhoun VD, Rachakonda S, Chen J, Patel V, Liu J (2015). Patterns of gray matter abnormalities in schizophrenia based on an international mega-analysis. Schizophr Bull.

[10] Lorenzetti V, Solowij N, Yücel M (2016). The role of cannabinoids in neuroanatomic alterations in cannabis users. Biol Psychiatry.

[11] Bangalore SS, Prasad KMR, Montrose DM, Goradia DD, Diwadkar VA, Keshavan MS (2008). Cannabis use and brain structural alterations in first episode schizophrenia-a region of interest, voxel based morphometric study. Schizophr Res.

[12] Buchy L, Mathalon DH, Cannon TD, Cadenhead KS, Cornblatt BA, McGlashan TH (2016). Relation between cannabis use and subcortical volumes in people at clinical high risk of psychosis. Psychiatry Res Neuroimaging.

[13] Batalla A, Bhattacharyya S, Yücel M, Fusar-Poli P, Crippa JA, Nogué S (2013). Structural and functional imaging studies in chronic cannabis users: a systematic review of adolescent and adult findings. PLoS ONE.

[14] Battistella G, Fornari E, Annoni J-M, Chtioui H, Dao K, Fabritius M (2014). Long-term effects of cannabis on brain structure. Neuropsychopharmacology.

[15] Wilson W, Mathew R, Turkington T, Hawk T, Coleman RE, Provenzale J (2000). Brain morphological changes and early marijuana use: a magnetic resonance and positron emission tomography study. J Addict Dis.

[16] Medina KL, McQueeny T, Nagel BJ, Hanson KL, Yang TT, Tapert SF (2009). Prefrontal cortex morphometry in abstinent adolescent marijuana users: subtle gender effects. Addict Biol.

[17] Weiland BJ, Thayer RE, Depue BE, Sabbineni A, Bryan AD, Hutchison KE (2015). Daily marijuana use is not associated with brain morphometric measures in adolescents or adults. J Neurosci.

[18] Chye Y, Christensen E, Yücel M (2019). Cannabis use in adolescence: a review of neuroimaging findings. J Dual Diagn..

[19] Rapp C, Bugra H, Riecher-Rössler A, Tamagni C, Borgwardt S (2012). Effects of cannabis use on human brain structure in psychosis: a systematic review combining in vivo structural neuroimaging and post mortem studies. Curr Pharm Des.

[20] Ashburner J, Friston KJ (2005). Unified segmentation. Neuroimage.

[21] Horwitz B (2003). The elusive concept of brain connectivity. Neuroimage.

[22] Greicius MD, Supekar K, Menon V, Dougherty RF (2009). Resting-state functional connectivity reflects structural connectivity in the default mode network. Cereb Cortex.

[23] Morgan SE, White SR, Bullmore ET, Vértes PE (2018). A network neuroscience approach to typical and atypical brain development. Biol Psychiatry Cogn Neurosci Neuroimaging.

[24] Evans AC (2013). Networks of anatomical covariance. Neuroimage.

[25] Alexander-Bloch A, Giedd JN, Bullmore E (2013). Imaging structural co-variance between human brain regions. Nat Rev Neurosci.

[26] Gupta CN, Turner JA, Calhoun VD (2019). Source-based morphometry: a decade of covarying structural brain patterns. Brain Struct Funct..

[27] Xu L, Groth KM, Pearlson G, Schretlen DJ, Calhoun VD (2009). Source-based morphometry: the use of independent component analysis to identify gray matter differences with application to schizophrenia. Hum Brain Mapp.

[28] Chen J, Liu J, Calhoun VD, Arias-Vasquez A, Zwiers MP, Gupta CN (2014). Exploration of scanning effects in multi-site structural MRI studies. J Neurosci Methods.

[29] Luo L, Xu L, Jung R, Pearlson G, Adali T, Calhoun VD (2012). Constrained source-based morphometry identifies structural networks associated with default mode network. Brain Connect.

[30] Lin Q-H, Liu J, Zheng Y-R, Liang H, Calhoun VD (2010). Semiblind spatial ICA of fMRI using spatial constraints. Hum Brain Mapp.

[31] Isvoranu A-M, Borsboom D, van Os J, Guloksuz S (2016). A network approach to environmental impact in psychotic disorder: brief theoretical framework. Schizophr Bull.

[32] Koutsouleris N, Kambeitz-Ilankovic L, Ruhrmann S, Rosen M, Ruef A, Dwyer DB (2018). Prediction models of functional outcomes for individuals in the clinical high-risk state for psychosis or with recent-onset depression: a multimodal, multisite machine learning analysis. JAMA Psychiatry.

[33] First MB, Spitzer RL, Gibbon M, Williams JB. Structured clinical interview for DSM-IV-TR axis I disorders, research version, patient edition. (SCID-I/P). New York, NY; 2002.

[34] Dilling H, Mombour W, Schmidt MH, Schulte-Markwort E. Internationale Klassifikation psychischer Störungen: ICD–10 Kapitel V (F) diagnostische Kriterien für Forschung und Praxis. 6th ed. Bern: Hogrefe; 2016.

[35] Mushquash C, O’Conner BP (2006). SPSS and SAS programs for generalizability theory analyses. Behav Res Methods.

[36] Du Y, Lin D, Yu Q, Sui J, Chen J, Rachakonda S (2017). Comparison of IVA and GIG-ICA in brain functional network estimation using fMRI data. Front Neurosci.

[37] R Core Team. R: A language and environment for statistical computing. Vienna, Austria: R Foundation for Statistical Computing; 2017.

[38] Kuznetsova A, Brockhoff PB, Christensen RHB (2017). lmerTest package: tests in linear mixed effects.. J Stat Softw..

[39] Jaeger BC, Edwards LJ, Das K, Sen PK (2016). An R2 statistic for fixed effects in the generalized linear mixed model. J Appl Stat.

[40] Deng MY, McAlonan GM, Cheung C, Chiu CPY, Law CW, Cheung V (2009). A naturalistic study of grey matter volume increase after early treatment in anti-psychotic naïve, newly diagnosed schizophrenia. Psychopharmacology.

[41] Dazzan P, Morgan KD, Orr K, Hutchinson G, Chitnis X, Suckling J (2005). Different effects of typical and atypical antipsychotics on grey matter in first episode psychosis: the AESOP study. Neuropsychopharmacology.

[42] Thayer RE, YorkWilliams S, Karoly HC, Sabbineni A, Ewing SF, Bryan AD (2017). Structural neuroimaging correlates of alcohol and cannabis use in adolescents and adults. Addiction.

[43] Gallardo-Ruiz R, Crespo-Facorro B, Setién-Suero E, Tordesillas-Gutierrez D (2019). Long-term grey matter changes in first episode psychosis: a systematic review. Psychiatry Investig.

[44] Genovese CR, Lazar NA, Nichols T (2002). Thresholding of statistical maps in functional neuroimaging using the false discovery rate. Neuroimage.

[45] Epskamp S, Cramer AO, Waldorp LJ, Schmittmann VD, Borsboom D. qgraph: Network visualizations of relationships in psychometric data. J Stat Softw. 2012;48:1.18.

[46] Kay SR, Fiszbein A, Opler LA (1987). The positive and negative syndrome scale (PANSS) for schizophrenia. Schizophr Bull.

[47] Epskamp S, Borsboom D, Fried EI (2018). Estimating psychological networks and their accuracy: a tutorial paper. Behav Res Methods.

[48] McGlashan TH, Miller TJ, Woods SW, Hoffman RE, Davidson L. Instrument for the assessment of prodromal symptoms and states. In: Miller T, Mednick SA, McGlashan TH, Libiger J, Johannessen JO, editors. Early Intervention in Psychotic Disorders. NATO Science Series (Series D: Behavioural and Social Sciences). Dordrecht: Springer, 2001. p. 135–149.

[49] Lorenzetti V, Lubman DI, Whittle S, Solowij N, Yücel M (2010). Structural MRI findings in long-term cannabis users: What do we know?. Subst Use Misuse.

[50] Orr C, Spechler P, Cao Z, Albaugh M, Chaarani B, Mackey S (2019). Grey matter volume differences associated with extremely low levels of cannabis use in adolescence. J Neurosci.

[51] Medina KL, Nagel BJ, Tapert SF (2010). Abnormal cerebellar morphometry in abstinent adolescent marijuana users. Psychiatry Res.

[52] Cousijn J, Wiers RW, Ridderinkhof KR, van den Brink W, Veltman DJ, Goudriaan AE (2012). Grey matter alterations associated with cannabis use: Results of a VBM study in heavy cannabis users and healthy controls. Neuroimage.

[53] Wang Y, Zuo C, Xu Q, Hao L. Cerebellar thickness changes associated with heavy cannabis use: a 3-year longitudinal study. Addict Biol. 23 June 2020:e12931. 10.1111/adb.12931. 10.1111/adb.12931 32575152

[54] Tiemeier H, Lenroot RK, Greenstein DK, Tran L, Pierson R, Giedd JN (2010). Cerebellum development during childhood and adolescence: a longitudinal morphometric MRI study. Neuroimage.

[55] Schoeler T, Kambeitz J, Behlke I, Murray R, Bhattacharyya S (2016). The effects of cannabis on memory function in users with and without a psychotic disorder: Findings from a combined meta-analysis. Psychol Med.

[56] Lenroot RK, Giedd JN (2008). The changing impact of genes and environment on brain development during childhood and adolescence: Initial findings from a neuroimaging study of pediatric twins. Dev Psychopathol.

[57] Hamilton I, Galdas P, Essex H (2015). Cannabis psychosis, gender matters. Adv Dual Diagn.

[58] Kim T, Lee K-H, Oh H, Lee TY, Cho KIK, Lee J (2018). Cerebellar structural abnormalities associated with cognitive function in patients with first-episode psychosis. Front Psychiatry.

[59] Moberget T, Doan NT, Alnæs D, Kaufmann T, Córdova-Palomera A, Lagerberg TV (2018). Cerebellar volume and cerebellocerebral structural covariance in schizophrenia: a multisite mega-analysis of 983 patients and 1349 healthy controls. Mol Psychiatry.

[60] Antonucci LA, Pergola G, Pigoni A, Dwyer D, Kambeitz-Ilankovic L, Penzel N (2019). A pattern of cognitive deficits stratified for genetic and environmental risk reliably classifies patients with schizophrenia from healthy control subjects. Biol Psychiatry..

[61] Bagot KS, Milin R, Kaminer Y (2015). Adolescent initiation of cannabis use and early-onset psychosis. Subst Abus.

[62] van Gastel WA, Wigman JTW, Monshouwer K, Kahn RS, van Os J, Boks MPM (2012). Cannabis use and subclinical positive psychotic experiences in early adolescence: findings from a Dutch survey. Addiction.

[63] Fergusson DM, Horwood LJ, Ridder EM (2005). Tests of causal linkages between cannabis use and psychotic symptoms. Addiction.

[64] Fortin J-P, Cullen N, Sheline YI, Taylor WD, Aselcioglu I, Cook PA (2018). Harmonization of cortical thickness measurements across scanners and sites. Neuroimage.

[65] Croft J, Heron J, Teufel C, Cannon M, Wolke D, Thompson A (2019). Association of trauma type, age of exposure, and frequency in childhood and adolescence with psychotic experiences in early adulthood. JAMA Psychiatry.

[66] Cassidy CM, Joober R, King S, Malla AK (2011). Childhood symptoms of inattention-hyperactivity predict cannabis use in first episode psychosis. Schizophr Res.

