# Supplementary material for: Association between age of cannabis initiation and gray matter covariance networks in recent onset psychosis
Source: Neuropsychopharmacology. 2021 Mar 3;46(8):1484–93. doi: 10.1038/s41386-021-00977-9 (PMC8209059; doi:10.1038/s41386-021-00977-9)
Supplement: Supplementary file 1 — Supplementary Material [file 41386_2021_977_MOESM1_ESM.docx]

**Supplementary materials for “Association between Age of Cannabis Initiation and Gray Matter Covariance Networks in Recent Onset Psychosis”**

**Table of Content**

[1. Inclusion/Exclusion criteria for participants with recent-onset psychosis (ROP) in the PRONIA and CIP study. 3](#_Toc57035208)

[2. Acquisition and preprocessing of sMRI 4](#_Toc57035209)

[3. G-theory based voxel selection of the Reference Components 5](#_Toc57035210)

[4. Goodness of fit and matching of COIs and RCs. 5](#_Toc57035211)

[5. Sanity checks - Outlier detection 5](#_Toc57035212)

[5.1 MRI-based outlier detection 5](#_Toc57035213)

[5.2 Clinical based outlier detection 7](#_Toc57035214)

[6. Exploratory Network Analysis - Stability Analyses 8](#_Toc57035215)

[7. Analyses restricted to patients with a schizophrenia spectrum disorder 8](#_Toc57035216)

[7.1 MRI-based outlier detection - SBM 9](#_Toc57035217)

[7.2 Correspondence with reference components 9](#_Toc57035218)

[7.3 Association between age of cannabis initiation and cerebellar components 10](#_Toc57035219)

[8. Analyses in sample restricted to male 10](#_Toc57035220)

[8.1 MRI-based outlier detection – SBM – male only 11](#_Toc57035221)

[8.2 Correspondence with reference components - male only 11](#_Toc57035222)

[8.3 Association between age of cannabis initiation and the cerebellar component - male only 12](#_Toc57035223)

[9. Neurocognition 12](#_Toc57035224)

[9.1 Test battery 12](#_Toc57035225)

[9.2 Harmonization of RAVLT and HVLT-R (verbal learning) 12](#_Toc57035226)

[9.3 Construction of the cognitive domains 13](#_Toc57035227)

[9.4 Correlation with age of cannabis initiation and cerebellar component (COI-9) 14](#_Toc57035228)

[10 Voxel-based morphometry: 14](#_Toc57035229)

[10.1 Voxel-based morphometry: Methods 14](#_Toc57035230)

[10.2 Voxel-based morphometry: Results 14](#_Toc57035231)

[Supplementary - Tables 15](#_Toc57035232)

[sTable 1 Assessments of CIP and PRONIA - Table is adapted from [4] 15](#_Toc57035233)

[sTable 2 DSM-IV Diagnoses 18](#_Toc57035234)

[sTable 3 Comparison between included and excluded ROP based on missing age of cannabis use initiation 19](#_Toc57035235)

[sTable 4 Demographics and Clinical Data correlated with the age of cannabis initiation 20](#_Toc57035236)

[sTable 5 sMRI protocol per Site 20](#_Toc57035237)

[sTable 6 Cognitive Test Battery (PRONIA and CIP) - Table adapted from [22] 21](#_Toc57035238)

[sTable 7 averaged z-scores of all 6 cognitive domains and the composite score correlated with the age of cannabis use initiation and the cerebellar component (COI-9) 22](#_Toc57035239)

[sTable 8 Demographic and Clinical Data in the sample restricted to schizophrenia spectrum disorder (SSD) 23](#_Toc57035240)

[sTable 9 Demographic and Clinical Data in the sample restricted to male 24](#_Toc57035241)

[sTable 10 Voxel based morphometry Analysis Results 25](#_Toc57035242)

[Supplementary - Figures 26](#_Toc57035243)

[sFigure 1 Design of PRONIA and CIP studies (Figure adapted from ^12^) 26](#_Toc57035244)

[sFigure 2 Substance Use Questionnaire 27](#_Toc57035245)

[sFigure 3 Distribution of age of initiation 28](#_Toc57035246)

[sFigure 4 Correlation between the components from the current study and the reference components 28](#_Toc57035247)

[sFigure 5 Flow Diagram - Inclusion based on Outlier 29](#_Toc57035248)

[sFigure 6 Results of bootstrapping the network 1000 times 30](#_Toc57035249)

[sFigure 7 Stability of edge weights testing by case dropping subset bootstrapping for the six-item network 31](#_Toc57035250)

[sFigure 8 Results of the VBM analysis – Correlation between age of cannabis initiation and GM volume 32](#_Toc57035251)

[Supplementary – References 33](#_Toc57035252)

# ***Inclusion/Exclusion criteria for participants with recent-onset psychosis (ROP) in the PRONIA and CIP study.***

ROP had to meet a DSM-IV diagnoses for an affective or non-affective psychotic episode in the last three months and no previous psychotic episode more than 24 months ago. For both studies, trained clinical raters made the PRONIA and CIP diagnoses based on SCID-1 interviews for DSM-IV disorders[1]. Additional medical records were consulted to aid the diagnostic process. All diagnoses were confirmed in weekly meetings with the principal investigators at each site. In the current study, we applied an additional inclusion criteria that ROP patients should have clinically relevant comorbid cannabis use, defined by a close temporal association between the onset of psychotic symptoms and initiation of cannabis use; i.e. cannabis use preceding the onset of psychotic symptoms by no more than two weeks, and/or by a lifetime cannabis abuse or dependency[1]. In the current analysis, participants were only included when the initiation age of the first cannabis use had been assessed. Participants from both studies were excluded if they had taken antipsychotic medication for more than 90 cumulative days at or above the minimum dosage indicated for first episode psychosis as specified in the DGPPN S3 Guidelines (guideline manual is available in

<https://www.dgppn.de/_Resources/Persistent/43ca38d4b003b8150b856df48211df68e412d9c9/038-009k_S3_Schizophrenie_2019-03.pdf>).

Further exclusion criteria were any traumatic head injury with loss of consciousness for more than five minutes, any contraindication for MRI, any neurological or somatic disease affecting the brain, a lifetime diagnoses of alcohol dependency, or inadequate language proficiency in English or the national language at the respective site.

# ***Acquisition and preprocessing of sMRI***

Data was acquired with isotropic or nearly isotropic voxel size, with a preferred voxel size of 1 mm^3^. The parameters of the field of view had to ensure full 3D coverage of the entire brain including the cerebellum, and other imaging parameters had to maximize the contrast between white matter and cortical ribbon as well as obtaining an optimal signal-to-noise ratio.

For pre-processing, we used the open-source CAT12 toolbox (version r1155;<http://dbm.neuro.uni-jena.de/cat12/>), an extension of SPM12 running in MATLAB 2018a. As a first step, all images were segmented into GM, WM and cerebrospinal fluid maps and normalized to stereotactic space of Montreal Neurological Institute (MNI-152 space). To derive GM volume maps, images were multiplied with the Jacobian determinants obtained during registration. *Post-hoc* quality checks were performed by correlating each slice across all subjects. Three scans deviated by more than two standard deviations (SD) of the mean, and were consequently re-examined visually. Due to artifacts, one image had to be excluded from the subsequent analysis, whereas the other two passed (sFigure 5 and 5 Sanity Checks). In a next step, we regressed age and sex effects voxel-wise, as these factors have an impact on GM, and previous studies have shown that regressing for their effects prior to SBM analysis makes the components more sensitive to group differences[2]. Subsequently, images were realigned to a two mm voxel resolution and smoothed with a ten mm (full-width at half maximum) Gaussian kernel[3].

# ***G-theory based voxel selection of the Reference Components***

MRI data from six individuals, who agreed to be scanned at seven of the eight included PRONIA sites (Munich, Milan Niguarda, Basel, Cologne, Birmingham, Turku, Udine), were analysed voxel-wise for subject- and site-specific variation. Here, higher g-values indicate high subject- and low site-specific variation, and low g-values indicate high site-specific and low subject specific variation[4]. The RCs were thresholded with the g-mask (voxels>0) using SPM12 running in MATLAB 2020 (Main Text, Figure 1).

# ***Goodness of fit and matching of COIs and RCs.***

To match the COIs derived from the current study with the RCs, we used a stepwise procedure as described previously[5]. To this end, we calculated the Pearson correlation coefficients (*r*) between all the RCs and all the COIs to derive a similarity matrix (sFigure 4). Based on this matrix, we defined the two components with the highest correlation as the first match. Next, we excluded the components of the first match, and correlated all remaining RCs and all remaining COIs with each other, where the RC and the COI with the highest correlation was considered as the second match. This procedure was repeated until all COIs were matched with the RCs, which was satisfied when four matched pairs of RCs and COIs were found. Based on previous study criteria[5], we considered as reliable a threshold of *r* > 0.5 between RCs and COIs.

# ***Sanity checks - Outlier detection***

## *5.1 MRI-based outlier detection*

Originally, we included 105 ROP cases from the PRONIA and CIP studies. We tested the validity of the sMRI in a stepwise procedure (see Flow diagram sFigure 5). We performed a three-step check to investigate for the presence of outliers based both on first-level and source-based MRI information. As a first step, we used the Computational Anatomy Toolbox[6] to get the volume-wise correlation of the unsmoothed sMRI images between all subjects. As a second step, we checked whether individuals with a high deviation from the average volume-wise correlation (defined as ± 2 SDs) presented any visual artifacts. Here, we inspected the raw images for presence of technical artifacts (e.g. blurring, ringing, wrapping, or incomplete head coverage). For segmented images we checked for general image quality (e.g. excessive noise, poor image contrast and poor boundaries). Scans from three individuals had a volume-wise correlation deviation by more than 2 SDs from the mean volume-wise correlation (*r* = 0.877). Following the instructions of the manual of the Computational Anatomy toolbox[7] these subjects were not simply removed from the analyses but were carefully checked. Of these, two scans (*r* = 0.865, *r* = 0.864) had no visual artefacts, while one scan (*r* = 0.857) had a scanner-related artefact, and was thus removed from all further analyses.

As a third step, to avoid single outliers with extremely high source variability that might otherwise drive spurious significant results, we also checked for outliers after the application of the GIG-ICA algorithm to the images smoothed with a 10-mm kernel. Hence, after creating the components of interest (COIs) in 104 individuals, we performed an additional component-wise quality control check employing Grubb’s test[8]. No outlier was found for COI-1 and COI-2 at a significance level of alpha = 0.05 (COI-1 (mean [SD]) = 0.0015 [0.0047], COI-2 (mean [SD]) = -0.0005 [0.0051]). One subject in COI-3 and one subject in COI-9 had significantly higher source variability (alpha <0.05). The outlier in COI-3 had a loading coefficient of -0.0249 (z = 3.673), which was significantly higher than the critical z-value (3.397) (COI-3 (mean [SD]) = -0.0064 [0.0050]). The outlier in COI-9 had a loading coefficient of 0.0219 (z = 4.7116), which was significantly higher than the critical z-value (3.397) (COI-9 (mean [SD]) = -0.0008 [0.0048]). We removed the two subjects with loading coefficients that deviated more than 2 SD from the mean. Subsequently, SBM was repeated and further analyses were calculated on this edited sample (n = 102).

## *5.2 Clinical based outlier detection*

To check the distribution of clinical data across the remaining 102 individuals, we investigated the potential presence of significant outliers for the factors *age of cannabis use initiation* and for the *duration of heaviest cannabis use*. After rechecking our data for typographic errors, we repeated our analyses with exclusion of significant outliers in age of cannabis initiation and duration of heaviest cannabis use. Results revealed that these outliers did not drive the significance of our findings, and furthermore their values were plausible (clinically speaking). Hence, all results in the paper are reported with inclusion of the outliers; here, we also present corresponding results with removal of all outliers.

Of the 102 remaining subjects, seven subjects were excluded because they were significant outliers with respect to age of initiation (> 26 years; N = 2), and duration of heaviest cannabis use (> 2390 days; N = 5), and another eight subjects were excluded due to missing data on duration of heaviest use. We repeated our analysis of differences in GMV covariation due to age of cannabis initiation employing a linear mixed effects model with the decreased sample size (N = 87). As before, loading coefficients entered the model as dependent variables, age of cannabis use initiation was used as a fixed effect, and site was a dummy-coded random effect. This analyses with a sample without outliers yielded comparable results to our original analysis. Significantly higher loading coefficient for COI-9 retained its association with an earlier initiation of cannabis use after correcting for site (t_84_ = -2.762, p_FDR_= 0.02). Adding several possible confounding factors (duration of heaviest use, chlorpromazine equivalent dosage cumulative lifetime, current antipsychotic intake (yes/no), alcohol abuse (yes/no), duration of illness) as additional fixed effect only slightly affected the results. Still, significantly higher loading coefficients in COI-9 were found to be associated with an earlier initiation (t_84_ = -2.89, p_FDR_= 0.02), while the with the difference that the random effect ‘site’ became significant in this model (t_80_ = 2.585, p_FDR_ = 0.04). No other components showed any significant effects with the initiation age. All *p*-values were corrected for multiple testing using the false discovery (FDR) with a threshold of p_FDR_<.05[9].

# ***Exploratory Network Analysis - Stability Analyses***

As recommended in current literature[10,11], we performed several robustness and stability analyses using bootstrapping from the R-package ‘bootnet’ version 1.3[11]. First, we bootstrapped our network 1000 times to derive 95 % bootstrapped confidence intervals for the edge weights. Additionally, we evaluated the number of bootstrapped networks in which each edge was set to zero, i.e. was not included in the network (sFigure 6). Furthermore, we tested the stability of our results by calculating the network with subsamples, dropping cases gradually, and correlated the resulting networks calculated for the subsamples with the original network (sFigure 7). The bootstrapped results indicating that the edges found in our network are relatively stable, but that decreasing the sample size does have some impact on our results.

# ***Analyses restricted to patients with a schizophrenia spectrum disorder***

To improve comparability between the sample from which the RCs were derived (Gupta et al., 2015[3]) and further reduce heterogeneity due to variety in diagnoses and severity of symptoms we repeated our analyses in individuals with DSM-IV diagnoses of schizophrenia spectrum; i.e. schizophrenia, schizophreniform disorder and schizoaffective disorder. Hence, we performed initial GIG-ICA on 47 subjects.

## ***7.1 MRI-based outlier detection - SBM***

Following our approach to control for extremely high source variability (Supplement 5.1), by a component-wise quality control check employing Grubb’s test[8] we included 44 subjects in our final analyses. Originally, we have performed the SBM analyses on 47 subjects. At a significance level of alpha = 0.05 no outlier was found for COI-2 and COI-9 (COI-2 (mean [SD]) = 0.0017 [0.0061], COI-9 (mean [SD]) = -0.0024 [0.0062]). One subject in COI-1 and 2 subjects in COI-3 had significantly higher or lower source variability (alpha <0.05). The outlier in COI-1 had a loading coefficient of 0.0176 (z = 3.1803), which was significantly higher than the critical z-value (z = 2.936) (COI-1 (mean [SD] = -0.0003 [0.0056]). In COI-3 the two outliers had a loading coefficient of -0.0334 (z = -3.5755) and -0.0305 (z = -3.5880), respectively. Both were lower than the critical z-value for COI-3 (z = -2.936). These outliers were removed from our sample and SBM and all subsequent analyses were performed on the remaining subjects (n = 44). See sTable 8 for demographic, substance use and clinical information of the subsample restricted to SSD divided in early (<17 years) and late (≥ 17 years) users.

## ***7.2 Correspondence with reference components***

While the assignment of our components between COIs and the RCs remained comparable to the original analyses including all subjects, the correlation between them dropped for all 4 components, which is most likely explained by the reduced sample size (only 46 % of the original subjects were included). For COI-9 (the cerebellar component) the correlation with RC-9 (*r* = .455, p<0.001) was now slightly below our original inclusion threshold (*r* *>* .5). However, we decided to include this component in the subsequent analyses as the correlation dropped only slightly below the threshold and further, the main goal of this comparison was to test whether the effect in this particular component (COI-9) would hold for SSD. COI-1 and COI-2 were also included in our analyses as they passed the threshold with *r* = .522 (p < 0.001) and *r* = .587 (p <0.001), respectively. COI-3 again did not pass our threshold *r* =.250 (p<0.001) and was thus excluded from all subsequent analyses.

## ***7.3 Association between age of cannabis initiation and cerebellar components***

Using the same model as in the whole sample, higher loading coefficients for COI-9 were again significantly associated with an earlier initiation age of cannabis use after correcting for multiple comparisons (t_44_=-2.543, p_FDR_=0.04). Like in the whole sample, in patients restricted to schizophrenia spectrum disorder (SSD) neither a significant effect of the initiation age of cannabis use was found for COI-1 (t_44_=-0.239, p_FDR_=0.98) nor for COI-2 (t_44_=-0.804, p_FDR_=0.38).

# ***Analyses in sample restricted to male***

The developmental trajectories of the cerebellum in males are protracted in comparison with females up to 5 years. Notably, disorders associated with cerebellar abnormalities, such as autism spectrum disorder[12] and attention-deficit/hyperactivity disorder[13] more likely occur in men. It has been hypothesized that this might partially be explained by the protracted and hence more vulnerable cerebellar development in male[14]. A study about admissions to National Health Service hospitals in England has found that the likelihood to develop psychosis is higher in men compared with women and that this phenomenon is even more pronounced in cannabis psychosis (male:female, 4:1)[15]. Following the reasoning, that later development of a brain area makes it more prone to harmful effects, this sexual dimorphism could at least be partially explained by cerebellar maturational differences. To test for a sex-specific association between the initiation of cannabis use and GMV we have repeated our analyses in the male subjects only.

## ***8.1 MRI-based outlier detection – SBM – male only***

Following our approach to control for extremely high source variability (Supplement 5.1), by a component-wise quality control check employing Grubb’s test [8] we included 79 subjects in our final analyses. Originally, we have performed the SBM analyses on 81 subjects. At a significance level of alpha = 0.05 no outlier were found for COI-2 and COI-4 (COI-2 (mean [SD]) = 0.00005 [0.0055], COI-4 (mean [SD]) = 0.0021 [0.0052]). One subject in COI-9 and 1 subject in COI-3 had significantly higher or lower source variability (alpha <0.05). The outlier in COI-9 had a loading coefficient of 0.0225 (z = 4.3488), which was significantly higher than the critical z-value (3.3106) (COI-9 (mean [SD] = -0.0007 [0.0053]). In COI-3 the outlier had a loading coefficient of -0.0245 (z = 3.5399) that was higher than the critical z-value (z = 3.3106) (COI-3 (mean [SD] = 0.0021 [0.0052]). These outliers were removed from our sample and SBM and all subsequent analyses were performed on the remaining subjects (n = 79). See sTable 9 for demographic, substance use and clinical information of the subsample restricted to male divided in early (<17 years) and late (≥ 17 years) users.

## ***8.2 Correspondence with reference components - male only***

The assignment of our components between COIs and the RCs remained similar to the original analyses including all subjects. A slightly lower correlation is most likely explained by the reduced sample size (only 77 % of the original subjects were included). As in the original analysis COI-9, COI-1 and COI-2 were included in our analyses, with correlations of *r* =.530 (p<0.001), *r* =.642 (p<0.001) and *r* =.664 (p<0.001), respectively. COI-3 again did not pass our threshold *r* =.332 (p<0.001) and was thus excluded from all subsequent analyses.

## ***8.3 Association between age of cannabis initiation and the cerebellar component - male only***

Using the same model as in the whole sample, higher loading coefficients for COI-9 were again significantly associated with an earlier initiation age of cannabis use after correcting for multiple comparisons (t_77_=2.336, p_FDR_=0.02). Like in the whole sample, in the male subjects alone neither a significant effect of the age of cannabis initiation was found for COI-1 (t_31_=-0.057, p_FDR_=0.75) nor for COI-2 (t_26_=-0.396, p_FDR_=0.43).

Unfortunately, our limited sample size of female subjects (N = 23) did not allow to test our model in a subsample restricted to female subjects.

# ***Neurocognition***

We further tested for associations between the age of cannabis use initiation and the neurocognitive performance, as previous literature indicates drug-related cognitive disturbances[16]. Additionally, we examined whether our finding of altered grey matter volume in the cerebellum might also be associated with cognitive disturbances as the cerebellum has recently been associated with higher cognitive functions, that evolve during adolescence[17].

## ***9.1 Test battery***

The same test battery was administered in PRONIA and CIP (see sTable 1).

## ***9.2 Harmonization of RAVLT and HVLT-R (verbal learning)***

While verbal learning in CIP as well as in most individuals from the PRONIA cohort was measured with the Rey auditory verbal learning test (RAVLT)[18], in one research site (Turku, N = 5) the Hopkins verbal learning test-revised HVLT-R[19] was used. These two tests assess the same concepts of verbal learning but for combining their results they have to be harmonized first. Thereby, it needs to be taken into account that they administer (i) a different number of items, 12 and 15 for HVLT-R and RAVLT, respectively, (ii) a different number of trials (3 for HVLT-R, 5 for RAVLT) and (iii) that the HVLT-R includes a subgrouping of semantic.

For the purpose of harmonization, 36 healthy controls from the PRONIA study at 5 different sites (Munich, Milan, Udine, Cologne and Birmingham) performed both tests, HVLT-R and RAVLT. Their mean age was 23.17 (SD 6.22) and 22 of them were male (61.1 %). All individuals performed the tests within at least 2 hours and the order of the tests was such that half of the subjects started with HVLT-R and the other half with RAVLT to account for habituation effects.

Based on the performance of these subjects, the following linear regression model was used to transform the HVLT-R data from participants from Turku to the RAVLT’s 5 repetitions:

RAVLT-sum5 = a*HVLT-Rsum3 + b

With a = 25.51264904

b = 1.191092045

## ***9.3 Construction of the cognitive domains***

Based on our cognitive test battery (see sTable 1 and sTable 6) we have built 6 of the 7 cognitive domains from the Measurement and Treatment Research to Improve Cognition in Schizophrenia (MATRICS [20,21]) recommendations; i.e. social cognition, working memory, speed of processing, verbal learning, reasoning and attention (see [22]). Verbal learning, one of the original cognitive domains from the MATRICS, could not be included in our analyses as neither the PRONIA nor the CIP study assessed a test comparable to the ones from the original MATRICS. While for some domains we have assessed the identical tests in the PRONIA/CIP neurocognitive battery as in the original MATRICS for other domains we had to replace them by comparable tests measuring the same construct (see sTable 6)

## ***9.4 Correlation with age of cannabis initiation and cerebellar component (COI-9)***

To test whether there was an association between age of cannabis use initiation or COI-9 with one of the cognitive domains we have correlated them with each other. However, all correlations were non-significant (see sTable 7). Hence, the initiation age of cannabis use initiation seemingly had no impact on the cognitive performance in recent onset psychosis with clinically relevant cannabis consumption.

# ***10 Voxel-based morphometry:***

## ***10.1 Voxel-based morphometry: Methods***

We performed a univariate VBM analysis to test for GMV correlations with the age of initiation of cannabis use. Our smoothed images were regressed voxel-wise for age and sex effects as well as for total intracranial volume. In the general linear model, age of cannabis use initiation was the independent variable and site was integrated as a dummy-coded covariate. Results were thresholded with FWE-corrected p < 0.05.

## ***10.2 Voxel-based morphometry: Results***

We did not find any significant volume difference associated with the age of initiation of cannabis use at the proposed threshold of FWE-p<0.05. At an uncorrected threshold (p < 0.005, *k* = 5) two clusters were significantly correlated with age of cannabis use initiation. The direction of this effect was the same as for the SBM analysis, i.e. more GM volume in the cerebellum was associated with an earlier initiation of cannabis use (sTable10 and sFigure8).

# ***Supplementary - Tables***

## ***sTable 1 Assessments of CIP and PRONIA - Table is adapted from*** ***[4]***

| **Instrument** | **Form** | **Baseline** |  | **IV3** | **IV6** | **T1** |  | **IV12** | **IV15** | **T2** |
| --- | --- | --- | --- | --- | --- | --- | --- | --- | --- | --- |
|  |  | **ROP** | **CIP** | **ROP** | **ROP** | **ROP** | **CIP** | **ROP** | **ROP** | **ROP** |
| **General data** | **OR** | **X** | **X** |  |  | **X** | **X** |  |  |  |
| **Reasons for referral** | **OR** | **X** | **X** |  |  |  |  |  |  |  |
| **Treatment documentation** | **OR** | **X** | **X** | **X** | **X** | **X** | **X** | **X** | **X** | **X** |
| **Somatic state and health history** | **OR** | **X** | **X** |  |  | **X** | **X** |  |  | **X** |
| **SPI-A COGDIS/ COPER****[23]** | **OR** | **X** |  | **X** | **X** | **X** |  | **X** | **X** | **X** |
| **SIPS positive symptoms****[24]** | **OR** | **X** | **X** | **X** | **X** | **X** | **X** | **X** | **X** | **X** |
| **CAARMS****[25]** | **OR** | **X** | **X** | **X** | **X** | **X** | **X** | **X** | **X** | **X** |
| **GAF****[26]** | **OR** | **X** | **X** | **X** | **X** | **X** | **X** | **X** | **X** | **X** |
| **UHR - Schizotypy, genetic risk** | **OR** | **X** | **X** | **X** |  | **X** | **X** |  |  | **X** |
| **CHR criteria** | **OR** | **X** |  |  |  | **X** |  |  |  | **X** |
| **SCID-IV screening****[1]** | **OR** | **X** | **X** |  |  | **X** | **X** |  |  | **X** |
| **SCID-IV summary****[1]** | **OR** | **X** | **X** |  |  | **X** | **X** |  |  | **X** |
| **Demographic and biographic data** | **OR** | **X** | **X** |  |  | **X** | **X** |  |  | **X** |
| **PAS****[27]** | **OR** | **X** | **X** |  |  | **X** | **X** |  |  | **X** |
| **SPI-A****[23]** | **OR** | **X** |  |  |  | **X** |  | **X** | **X** | **X** |
| **SIPS negative, disorganized and general symptoms****[24]** | **OR** | **X** | **X** |  |  | **X** | **X** |  |  | **X** |
| **PANSS****[28]** | **OR** | **X** | **X** | **X** | **X** | **X** | **X** | **X** | **X** | **X** |
| **SANS****[29]** | **OR** | **X** | **X** |  |  | **X** | **X** |  |  | **X** |
| **Chart of life events** | **OR** | **X** | **X** | **X** | **X** | **X** | **X** | **X** | **X** | **X** |
| **FROGS****[30]** | **OR** | **X** | **X** |  |  | **X** | **X** |  |  | **X** |
| **GF: Social & role****[31]** | **OR** | **X** | **X** | **X** | **X** | **X** | **X** | **X** | **X** | **X** |
| **Prognostic evaluation** | **OR** | **X** | **X** |  |  | **X** | **X** |  |  | **X** |
| **Substance use questionnaire** | **OR** | **X** | **X** | **X** | **X** | **X** | **X** | **X** | **X** | **X** |
| **MSPSS****[32]** | **SR** | **X** | **X** |  |  | **X** | **X** |  |  | **X** |
| **RSA****[33]** | **SR** | **X** | **X** |  |  | **X** | **X** |  |  | **X** |
| **CISS 24****[34]** | **SR** | **X** | **X** |  |  | **X** | **X** |  |  | **X** |
| **SPIN****[35]** | **SR** | **X** | **X** |  |  | **X** | **X** |  |  | **X** |
| **BDI-II****[36]** | **SR** | **X** | **X** | **X** | **X** | **X** | **X** | **X** | **X** | **X** |
| **WHO-QOL-BREF****[37]** | **SR** | **X** | **X** |  |  | **X** | **X** |  |  | **X** |
| **EHI-SR****[38]** | **SR** | **X** | **X** |  |  |  |  |  |  |  |
| **LEE****[39]** | **SR** | **X** | **X** |  |  | **X** | **X** |  |  | **X** |
| **Wisconsin scales****[40]** | **SR** | **X** | **X** |  |  |  |  |  |  |  |
| **EDS****[41]** | **SR** | **X** | **X** |  |  |  |  |  |  |  |
| **Bullying scale** **[42]** | **SR** | **X** | **X** |  |  |  |  |  |  |  |
| **CTQ****[43]** | **SR** | **X** | **X** |  |  |  |  |  |  |  |
| **NEO-FFI****[44]** | **SR** | **X** | **X** |  |  |  |  |  |  |  |
| **Substance use** | **SR** |  | **X** |  |  |  |  |  |  |  |
| **Cannabis experience questionnaire (CEQ)** | **SR** |  | **X** |  |  |  |  |  |  |  |
| **Severity of dependency scale (SES)** | **SR** |  | **X** |  |  |  |  |  |  |  |
| **DS backward (BACS)** | **NPT** | **X** | **X** |  |  | **X** |  |  |  |  |
| **DS forward (BACS)** | **NPT** | **X** | **X** |  |  | **X** |  |  |  |  |
| **CPT-IP (BACS)****[45]** | **NPT** | **X** | **X** |  |  | **X** |  |  |  |  |
| **DANVA****[46]** | **NPT** | **X** | **X** |  |  | **X** |  |  |  |  |
| **DSST** | **NPT** | **X** | **X** |  |  | **X** |  |  |  |  |
| **RAVLT*****[18]** | **NPT** | **X** | **X** |  |  | **X** |  |  |  |  |
| **ROCF****[47]** | **NPT** | **X** | **X** |  |  | **X** |  |  |  |  |
| **SAT****[48]** | **NPT** | **X** | **X** |  |  | **X** |  |  |  |  |
| **SOPT****[49]** | **NPT** | **X** | **X** |  |  | **X** |  |  |  |  |
| **TMT-A****[50]** | **NPT** | **X** | **X** |  |  | **X** |  |  |  |  |
| **TMT-B****[50]** | **NPT** | **X** | **X** |  |  | **X** |  |  |  |  |
| **VF phonetic** | **NPT** | **X** | **X** |  |  | **X** |  |  |  |  |
| **VF semantic** | **NPT** | **X** | **X** |  |  | **X** |  |  |  |  |
| **WAIS-III****[51]** | **NPT** | **X** | **X** |  |  | **X** |  |  |  |  |
| **sMRI** | **MRI** | **X** | **X** |  |  | **X** |  |  |  |  |
| **rs-fMRI** | **MRI** | **X** | **X** |  |  | **X** |  |  |  |  |
| **DWI** | **MRI** | **X** | **X** |  |  | **X** |  |  |  |  |
| **blood sample** | **bio** | **X** |  |  |  | **X** |  |  |  |  |
| **hair sample** | **THC** |  | **X** |  |  |  |  |  |  |  |
| **Urine sample** | **THC** |  | **X** |  |  |  |  |  |  |  |
| **EEG** | **EEG** |  | **X** |  |  |  |  |  |  |  |

*Abbreviation:* IV3 = interval three months after baseline, IV6 = interval six months after baseline, T1 = interval nine months after baseline, IV12 = interval 12 months after baseline, IV15 = interval 15 months after baseline, T2 = interval 18 months after baseline, OR = Observer-based-rating instrument, SR = Self-rating-based instrument, NPT = Neuropsychological Test, MRI = Magnetic Resonance Imaging, sMRI = structural Magnetic Resonance Imaging, rs-fMRI = resting-state functional Magnetic Resonance Imaging, DWI = Diffusion Weighted Imaging, bio = biological test, THC = Cannabis-related test, EEG = Electro Encephalography, SPI-A COGDIS/COPER = Schizophrenia Proneness Instrument - Cognitive disturbances / Cognitive-Perceptual disturbances, CAARMS = Comprehensive Assessment of the At-Risk Mental States, CHR Criteria, SIPS = Standardized Interview for the assessment of Prodromal Symptoms (modified version 5.0), GAF = Global Assessment of Functioning, UHR – Schizotypy, genetic risk = Genetic Risk Interview for the Assessment of Schizotypal personality traits, and familial risk for psychosis, CHR criteria = Clinical High-Risk criteria summary questionnaire, SCID-IV Screening/Summary = Structured Clinical Interview for DSM-IV, PAS = Premorbid Adjustment Scale, PANSS = Positive and Negative Syndrome Scale, SANS = Scale for the Assessment of Negative Symptoms, FROGS = Functional Remission in General Schizophrenia, GF: Social/Role = Global Functioning: Social/Role, MSPSS = the Multidimensional Scale for Perceived Social Support, RSA = Resilience Scale for Adults, CISS 24 = Coping Inventory for Stressful Situations – 24 items, SPIN = Social Phobia Inventory, BDI-II = Beck Depression Inventory II, WHO-QOL-BREF = WHO Quality of Life Questionnaire – Brief Version, EHI-SR = Edinburgh Handedness Inventory – Short Version, LEE = Level of Expressed Emotions, Wisconsin scales = , EDS = Everyday Discrimination Scale – Modified Version, CTQ = Childhood Trauma Questionnaire, NEO-FFI = NEO Five Factor Inventory of Personality Traits, DS = Auditory Digit Span (Forward/Backward) adapted from the PEBL battery, CPT-IP (BACS) = Continuous-Performance Test – Identical Pairs (adapted tablet version), DANVA = Diagnostic Analysis of

Non-Verbal Accuracy 2 (adapted tablet version), DSST = Digit-Symbol-Substitution Test from the BACS battery, RAVLT = , ROCF = Rey-Osterrieth complex figure, SAT = Salience Attribution Task (adapted version), SOPT = self-ordered pointing task (adapted version), TMT-A/-B = Trail-Making Test A and B, VF phonetic/semantic = verbal fluency test, WAIS-III = Wechsler Adult Intelligence Scale (3^rd^ edition)

*** in one of the research sites (Turku) the revised version of the Hopkins Verbal Learning Test (HVLT-R) was included instead of the RAVLT that was not available in Finnish. See the description of the two scales in 8. Neurocognition.**

## ***sTable 2 DSM-IV Diagnoses***

| **Variables** | **Early (< 17)** | **Late (17 +)** | **df** | **T/Z/x^2^** | **p-value** |
| --- | --- | --- | --- | --- | --- |
| **Samples and study variables** | | | | | |
| **Sample sizes** | 58 | 44 |  |  |  |
| **Substance use disorder** | | | | | |
| **Lifetime history of DSM-IV alcohol use disorder [N (%)]** | | | 1 | 0.002 | 0.967 |
| **Alcohol abuse (%)** | 13 (22.41) | 9 (20.45) |  |  |  |
| **Alcohol dependency (%)** | 0 (0) | 0 (0) |  |  |  |
| **Lifetime history of DSM-IV sedative-hypnotic-anxiolytic use disorder [N (%)]** | | | 0 | 0 | 1 |
| **Sedative-hypnotic-anxiolytic abuse (%)** | 1 (0) | 1 (0) |  |  |  |
| **Sedative-hypnotic-anxiolytic dependency (%)** | 0 (0) | 0 (0) |  |  |  |
| **Lifetime history of DSM-IV stimulants use disorder [N (%)]** | | | 1 | 1.002 | 0.293 |
| **Stimulants abuse (%)** | 7 (12.07) | 2 (4.55) |  |  |  |
| **Stimulants dependency (%)** | 0 (0) | 0 (0) |  |  |  |
| **Lifetime history of DSM-IV opoid use disorder [N (%)]** | | | 1 | 0 | 1 |
| **Opoid abuse (%)** | 2 (3.45) | 1 (2.27) |  |  |  |
| **Opoid dependency (%)** | 0 (0) | 0 (0) |  |  |  |
| **Lifetime history of DSM-IV cocaine use disorder [N (%)]** | | | 2 | 0.784 | 0.676 |
| **Cocaine abuse (%)** | 4 (6.90) | 3 (6.82) |  |  |  |
| **Cocaine dependency (%)** | 1 (1.72) | 0 (0) |  |  |  |
| **Lifetime history of DSM-IV hallucinogenes use disorder [N (%)]** | | | 1 | 0 | 0.991 |
| **Hallucinogenes abuse (%)** | 4 (6.90) | 4 (9.09) |  |  |  |
| **Hallucinogenes dependency (%)** | 0 (0) | 0 (0) |  |  |  |
| **Psychotic disorder** | | | | | |
| **Lifetime history of DSM-IV psychotic disorder [N (%)]** | | | 8 | 12.05 | 0.115 |
| **Schizophrenia** | 16 (15.7) | 11 (25) |  |  |  |
| **Schizophreniform disorder** | 7 (12.1) | 5 (8.6) |  |  |  |
| **Brief psychotic disorder** | 6 (10.3) | 2 (4.5) |  |  |  |
| **Schizoaffective disorder** | 5 (8.6) | 2 (4.5) |  |  |  |
| **Delusional disorder** | 4 (6.9) | 2 (4.5) |  |  |  |
| **Substance-induced psychotic disorder** | 17 (29.3) | 13 (29.6) |  |  |  |
| **Psychotic disorder not otherwise specified** | 0 (0) | 7 (15.9) |  |  |  |
| **Major depressive disorder with psychotic**  **symptoms** | 2 (3.5) | 2 (4.5) |  |  |  |
| **Bipolar disorder with psychotic symptoms** | 1 (1.7) | 0 (0) |  |  |  |

##

## ***sTable 3 Comparison between included and excluded ROP based on missing age of cannabis use initiation***

|  | **Missing** | **Not Missing** | **df** | **T/Z/X^2^** | **p-value** |
| --- | --- | --- | --- | --- | --- |
| **Samples and Study Variables** | | | | | |
| Sample sizes | 14 | 105 |  |  |  |
| CIP (%) | 0 (0) | 46 (43.8) | 1 | 8.236 | 0.004 |
| Age [mean (SD) years] | 23.8 (5.1) | 23.4 (4.2) | 15.44 | -0.282 | 0.782 |
| Sex [F (%)] | 3 (21.4) | 23 (21.9) | 1 |  | 1 |
| **Sample Size per Site** | | | 18 |  | <0.001 |
| Munich (%) | 1 (7.1) | 72 (68.6) |  |  |  |
| Milan Niguarda (%) | 0 (0) | 6 (5.7) |  |  |  |
| Basel (%) | 2 (14.3) | 0 (0) |  |  |  |
| Cologne (%) | 7 (50) | 5 (4.8) |  |  |  |
| Birmingham (%) | 0 (0) | 3 (2.9) |  |  |  |
| Turku (%) | 0 (0) | 5 (4.8) |  |  |  |
| Udine (%) | 0 (0) | 1 (1.0) |  |  |  |
| Düsseldorf (%) | 0 (0) | 2 (1.9) |  |  |  |
| Bari (%) | 2 (14.3) | 0 (0) |  |  |  |
| **Cannabis Use** | | | | | |
| Lifetime History of DSM-IV Cannabis Use Disorder [N (%)] | | | 6 |  | 0.047 |
| Cannabis abuse (%) | 12 (85.7) | 48 (45.7) |  |  |  |
| Cannabis dependency (%) | 2 (14.3) | 42 (40.0) |  |  |  |
| Cumulative months lifetime [mean (SD) months] | 24.5 (33.2) | 46.9 (38.7) | 1.09 | -0.933 | 0.511 |
| Duration of heaviest use [mean (SD) days] | 401.7 (693.7) | 620.7 (715.5) | 3.272 | -0.618 | 0.577 |
| **Level of Use in the Heaviest Use Period (%)** | | | 2 | 5.922 | 0.052 |
| > 10 times per month / dependency | 7 (50.0) | 86 (82.9) |  |  |  |
| < 10 times per month | 3 (21.4) | 11 (10.5) |  |  |  |
| Only once | 2 (14.3) | 4 (3.8) |  |  |  |
| Duration since last use [mean (SD) days] | 1889.8 (2514.2) | 315.5 (959.0) | 5.088 | 1.496 | 0.194 |
| **Level of Use in the Last 3 Months – Cumulative Frequency (%)** | | | 7 | 7.110 | 0.418 |
| 0 times | 5 (35.7) | 33 (31.4) |  |  |  |
| 1-5 times | 0 (0) | 8 (7.2) |  |  |  |
| 6-10 times | 0 (0) | 6 (5.7) |  |  |  |
| 11-15 times | 0 (0) | 5 (4.8) |  |  |  |
| 16-20 times | 0 (0) | 4 (3.8) |  |  |  |
| 21-30 times | 0 (0) | 3 (2.9) |  |  |  |
| > 30 times | 0 (0) | 21 (20.0) |  |  |  |
| **Psychopathology [mean (SD)]** | | | | | |
| Positive and Negative Syndrome Scale - Positive | 23.5 (5.1) | 19.37 (6.2) | 18.841 | 2.754 | 0.013 |
| Positive and Negative Syndrome Scale - Negative | 21.6 (7.5) | 14.5 (5.5) | 15.098 | 3.440 | 0.004 |
| Positive and Negative Syndrome Scale - General | 44.8 (12.3) | 34.2 (8.2) | 14.745 | 3.138 | 0.007 |
| Onset Age of Psychotic Disorder | 23.2 (5.2) | 23.5 (4.2) | 12.742 | -0.227 | 0.824 |
| **Medication [mean (SD)]** | | | | | |
| Currently treated (%) | 6 (42.9) | 66 (62.9) | 4 | - | 0.154 |
| Chlorpromazine equivalent (cumulative lifetime) | 5289.5 (7497.4) | 4728.9 (7548.6) | 12.315 | 0.235 | 0.818 |

## ***sTable 4 Demographics and Clinical Data correlated with the age of cannabis initiation***

|  | **Mean**  **(SD)** | **df** | **correlation [confidence interval]** | **p-value** |
| --- | --- | --- | --- | --- |
| Age (years) | 23.8 (4.1) | 100 | 0.090 [-0.107, 0.279] | 0.369 |
| Cumulative months lifetime (months) | 45.6 (38.6) | 56 | -0.359 [-0.565, -0.111] | 0.006 |
| Duration of heaviest use (days) | 624.3 (722.6) | 92 | -0.250 [-0.430, -0.050] | 0.015 |
| Duration since last use (days) | 330.3 (973.3) | 91 | -0.076 [-0.276, 0.129] | 0.468 |
| Level of Use in the Last 3 Months – Cumulative Frequency (%) | - |  | -0.014 | 0.907 |
| Level of Use in the Last 3 Months – Average Frequency | - |  | 0.140 | 0.255 |
| Positive and Negative Syndrome Scale - Positive | 19.5 (6.2) | 93 | -0.223 [-0.406, -0.023] | 0.030 |
| Positive and Negative Syndrome Scale - Negative | 14.5 (5.6) | 92 | 0.039 [-0.165, 0.240] | 0.712 |
| Positive and Negative Syndrome Scale - General | 34.2 (8.3) | 91 | -0.121 [-0.317, 0.085] | 0.248 |
| Onset Age of Psychotic Disorder | 23.5 (4.0) | 96 | 0.137 [-0.063, 0.327] | 0.178 |
| Chlorpromazine equivalent (cumulative lifetime) | 4824.4 (7594.6) | 97 | 0.186 [-0.012, 0.370] | 0.065 |

##

## ***sTable 5 sMRI protocol per Site***

| **PRONIA Site** | **Model** | **Field Strength** | **Flip Angle** | **TR (ms)** | **TE (ms)** | **Voxel size [mm]** | **FOV** | **Slice Number** |
| --- | --- | --- | --- | --- | --- | --- | --- | --- |
| Munich | Philips Ingenia | 3T | 8 | Shortest (9.4) | Shortest (5.5) | 0.97 x 0.97 x 1.0 | 250 x 250 | 190 |
| Milan Niguarda | Philips Achieva Intera | 1.5T | 12 | Shortest (8.1) | Shortest (3.7) | 0.94 x 0.94 x 1.0 | 240 x 240 | 170 |
| Basel | SIEMENS Verio / Prisma | 3T | 8 | 2000 | 3.4 | 1.0 x 1.0 x 1.0 | 256 x 256 | 176 |
| Cologne | Philips Achieva | 3T | 8 | 9.5 | 5.5 | 0.97 x 0.97 x 1.0 | 250 x 250 | 190 / 165 |
| Birming-  ham | Philips Achieva | 3T | 8 | 8.4 | 3.8 | 1.0 x 1.0 x 1.0 | 288 x 288 | 175 |
| Turku | Philips Ingenuity | 3T | 7 | 8.1 | 3.7 | 1.0 x 1.0 x 1.0 | 256 x 256 | 176 |
| Udine | Philips Achieva | 3T | 12 | Shortest (8.1) | Shortest (3.7) | 0.93 x 0.93 x 1.0 | 240 x 240 | 170 |
| Düsseldorf | SIEMENS TrioTim | 3T | 8 | 2000 | 3.4 | 1.0 x 1.0 x 1.0 | 256 x 256 | 176 |

## ***sTable 6 Cognitive Test Battery (PRONIA and CIP) - Table adapted from*** ***[22]***

| **Neurocognitive domain** | **Cognitive test** | **Description part of the test parts relevant for our analyses** | **Measure of interest** |
| --- | --- | --- | --- |
| Social cognition | Diagnostic Analysis of Nonverbal Accuracy -2 | Participants are presented with 24 faces on a tablet showing 4 different emotions; happy, neutral, angry, sad and have to decide which emotion is represented. | number of correct responses |
| Speed of processing | 1. Trail Making Test (TMT): Part A  2. Verbal Fluency: semantic  Wechsler Adult Intelligence Scale, 3^rd^ ed.,  3. digit symbol coding task | 1. participants have to combine numbers in ascendent order (paper-pencil)  2. participants had 1 minute to produce as many words as possible from the semantic category *animals*.  3. Participants were presented with 9 symbols each corresponding to a number from 1-9 on the top of a sheet of paper. Then, they had to write the corresponding number under as many symbols as possible in 1 minute on the same sheet of paper. (paper-pencil) | 1. time of execution  2. correct words  3. number of correctly matched symbols |
| Working memory | Wechsler Memory Scale, 3rd ed., spatial span subtest | Participants have to repeat sequences of numbers with increasing difficulties (one number added in each sequence) first forward then backward. | sum of number of correct trials |
| Verbal learning | Rey Auditory Verbal Learning Test (RAVLT) and for Turku harmonized HVLT-R (see harmonization description in 8.3) | Participants have to immediately recall as many words as possible from a list of 12 words that is audio-played to them. | sum of correctly recalled words |
| Reasoning | Wechsler Adult Intelligence Scale, 4th ed., Matrix Reasoning | Participants are presented with a matrix showing a sequence of abstract pictures. The participants have to decide which picture of a number of possible options would complete the sequence best. | sum of correct responses |
| Attention | Continuous Performance Task – Identical Pairs (CPT-IP) | Participants were presented with 300 four-digit numbers on a tablet-screen with a rate of one per second and had to click as fast as possible on a computer-mouse in case of identical repeating numbers. | difference between standardized z-scores of correct and false alarm |
| Global cognition | Composite across all cognitive measures included above (average z-score) | The scores from all included domains were standardized to z-scores. | Sum of all standardized z-scores. |

##

## ***sTable 7 averaged z-scores of all 6 cognitive domains and the composite score correlated with the age of cannabis use initiation and the cerebellar component (COI-9)***

|  | **df** | **correlation [confidence interval]** | **p-value** |
| --- | --- | --- | --- |
| **Age of cannabis use initiation** | | | |
| Social cognition | 94 | 0.043 [-0.159, 0.241] | 0.680 |
| Speed of processing | 91 | -0.096 [-0.294, 0.110] | 0.359 |
| Working memory | 94 | -0.109 [0.302, 0.094] | 0.292 |
| Verbal learning | 94 | -0.057 [-0.255, 0.145] | 0.580 |
| Reasoning | 90 | -0.087 [-0.287, 0.120] | 0.410 |
| Attention | 94 | 0.014 [-0.187, 0.214] | 0.890 |
| Global cognition | 88 | -0.094 [-0.295, 0.115] | 0.378 |
| **Loadings of cerebellar component (COI-9)** | | | |
| Social cognition | 94 | -0.028 [-0.228, 0.173] | 0.783 |
| Speed of processing | 91 | 0.048 [-0.157, 0.249] | 0.647 |
| Working memory | 94 | 0.046 [-0.156, 0.244] | 0.655 |
| Verbal learning | 94 | 0.072 [-0.131, 0.268] | 0.488 |
| Reasoning | 90 | -0.089 [-0.289, 0.118] | 0.398 |
| Attention | 94 | 0.074 [-0.128, 0.271] | 0.472 |
| Global cognition | 88 | 0.057 [-0.152, 0.261] | 0.594 |

##

## ***sTable 8 Demographic and Clinical Data in the sample restricted to schizophrenia spectrum disorder (SSD)***

|  | **Early (< 17)** | **Late (17 +)** | **df** | **T/Z/X^2^** | **p-value** |
| --- | --- | --- | --- | --- | --- |
| **Samples and Study Variables** | | | | | |
| Sample sizes | 27 | 17 |  |  |  |
| CIP (%) | 8 (29.6) | 2 (11.8) | 1 | 1.015 | 0.314 |
| Age [mean (SD) years] | 24.5 (4.7) | 24.0 (4.2) | 37.3 | 0.314 | 0.755 |
| Sex [F (%)] | 6 (22.2) | 5 (29.4) | 4 | - | 0.724 |
| **Sample Size per Site** | | | 12 | - | 0.1953 |
| Munich (%) | 19 (70.4) | 10 (58.8) |  |  |  |
| Milan Niguarda (%) | 0 (0) | 0 (0) |  |  |  |
| Basel (%) | 6 (22.2) | 2 (11.8) |  |  |  |
| Cologne (%) | 1 (3.7) | 3 (17.6) |  |  |  |
| Birmingham (%) | 1 (3.7) | 0 (0) |  |  |  |
| Turku (%) | 0 (0) | 1 (5.9) |  |  |  |
| Udine (%) | 0 (0) | 1 (5.9) |  |  |  |
| Düsseldorf (%) | 0 (0) | 0 (0) |  |  |  |
| **Cannabis Use** | | | | | |
| Lifetime History of DSM-IV Cannabis Use Disorder [N (%)] | | | 6 | - | 0.703 |
| Cannabis abuse (%) | 17 (63.0) | 13 (76.5) |  |  |  |
| Cannabis dependency (%) | 7 (25.9) | 3 (17.6) |  |  |  |
| Initiation Age [mean (SD) years] | 14.2 (1.2) | 20.8 (4.1) | 17.921 | -6.126 | <0.001 |
| Cumulative months lifetime [mean (SD) months] | 50 (34.5) | 19 (24.1) | 13.346 | 2.338 | 0.036 |
| Duration of heaviest use [mean (SD) days] | 589.1 (754.5) | 438.6 (484.7) | 35.960 | 0.755 | 0.455 |
| **Level of Use in the Heaviest Use Period (%)** | | | 2 | 2.053 | 0.358 |
| > 10 times per month / dependency | 22 (81.5) | 13 (76.5) |  |  |  |
| < 10 times per month | 2 (7.4) | 2 (11.8) |  |  |  |
| Only once | 3 (11.1) | 0 (0) |  |  |  |
| Duration since last use [mean (SD) days] | 582.6 (1499.3) | 523.7 (989.0) | 38.933 | 0.151 | 0.880 |
| **Level of Use in the Last 3 Months – Cumulative Frequency (%)** | | | 12 | - | 0.709 |
| 0 times | 11 (40.7) | 7 (41.2) |  |  |  |
| 1-5 times | 2 (7.4) | 3 (17.6) |  |  |  |
| 6-10 times | 1 (3.7) | 1 (5.9) |  |  |  |
| 11-15 times | 0 (0) | 0 (0) |  |  |  |
| 16-20 times | 1 (3.7) | 0 (0) |  |  |  |
| 21-30 times | 5 (18.5) | 1 (5.9) |  |  |  |
| > 30 times | 1 (3.7) | 2 (11.8) |  |  |  |
| **Psychopathology [mean (SD)]** | | | | | |
| Positive and Negative Syndrome Scale - Positive | 20.2 (5.6) | 17.4 (8.1) | 23.760 | 1.214 | 0.237 |
| Positive and Negative Syndrome Scale - Negative | 16.1 (5.5) | 15.9 (5.7) | 28.228 | 0.115 | 0.909 |
| Positive and Negative Syndrome Scale - General | 36.3 (7.8) | 33.5 (8.1) | 28.520 | 1.082 | 0.288 |
| Onset Age of Psychotic Disorder | 24.0 (4.9) | 23.8 (4.0) | 38.771 | 0.148 | 0.883 |
| Years between first cannabis use initiation and attenuated psychotic symptoms – years [mean (SD)] | 8.7 (5.5) | 4.4 (3.2) | 24.828 | 2.655 | 0.014 |
| Years between initiation of heaviest cannabis use and attenuated psychotic symptoms – years [mean (SD)] | 3.6 (5.0) | 2.0 (3.1) | 30.395 | 1.149 | 0.260 |
| **Medication [mean (SD)]** | | | | | |
| Currently treated (%) | 20 (74.1) | 9 (52.9) | 1 | 0.755 | 0.385 |
| Chlorpromazine equivalent (cumulative lifetime) | 4060.0 (5961.3) | 5656.8 (9130.1) | 22.698 | -0.625 | 0.538 |

## ***sTable 9 Demographic and Clinical Data in the sample restricted to male***

|  | **Early (< 17)** | **Late (17 +)** | **df** | **T/Z/X^2^** | **p-value** |
| --- | --- | --- | --- | --- | --- |
| **Samples and Study Variables** | | | | | |
| Sample sizes | 47 | 32 |  |  |  |
| CIP (%) | 24 (51.2) | 13 (40.6) | 1 | 0.467 | 0.495 |
| Age [mean (SD) years] | 23.5 (3.5) | 23.4 (4.3) | 57.124 | 0.121 | 0.904 |
| **Sample Size per Site** | | | 16 | - | 0.112 |
| Munich (%) | 35 (74.5) | 20 (62.5) |  |  |  |
| Milan Niguarda (%) | 0 (0) | 3 (9.4) |  |  |  |
| Basel (%) | 7 (14.9) | 2 (6.3) |  |  |  |
| Cologne (%) | 2 (4.3) | 2 (6.3) |  |  |  |
| Birmingham (%) | 3 (6.4) | 0 (0) |  |  |  |
| Turku (%) | 0 (0) | 3 (9.4) |  |  |  |
| Udine (%) | 0 (0) | 1 (3.1) |  |  |  |
| Düsseldorf (%) | 0 (0) | 1 (3.1) |  |  |  |
| **Cannabis Use** | | | | | |
| Lifetime History of DSM-IV Cannabis Use Disorder [N (%)] | | | 6 | - | 0.519 |
| Cannabis abuse (%) | 19 (40.4) | 16 (50.0) |  |  |  |
| Cannabis dependency (%) | 22 (46.8) | 11 (34.4) |  |  |  |
| Initiation Age [mean (SD) years] | 14.9 (1.1) | 17.8 (3.5) | 35.339 | -7.615 | <0.001 |
| Cumulative months lifetime [mean (SD) months] | 60.8 (39.6) | 25.4 (26.9) | 41.218 | 3.588 | <0.001 |
| Duration of heaviest use [mean (SD) days] | 847.5 (901.1) | 402.4 (428.9) | 65.403 | 2.850 | 0.006 |
| **Level of Use in the Heaviest Use Period (%)** | | | 2 | 0.687 | 0.709 |
| > 10 times per month / dependency | 41 (87.2) | 27 (84.4) |  |  |  |
| < 10 times per month | 4 (8.5) | 3 (9.4) |  |  |  |
| Only once | 1 (2.1) | 0 (0) |  |  |  |
| Duration since last use [mean (SD) days] | 275.0 (752.2) | 257.9 (729.8) | 59.092 | 0.096 | 0.924 |
| **Level of Use in the Last 3 Months – Cumulative Frequency (%)** | | | 16 | - | 0.870 |
| 0 times | 12 (25.5) | 9 (28.1) |  |  |  |
| 1-5 times | 2 (4.3) | 3 (9.4) |  |  |  |
| 6-10 times | 4 (8.5) | 1 (3.1) |  |  |  |
| 11-15 times | 3 (6.4) | 2 (6.3) |  |  |  |
| 16-20 times | 1 (2.1) | 2 (6.3) |  |  |  |
| 21-30 times | 2 (4.3) | 1 (3.1) |  |  |  |
| > 30 times | 11 (23.4) | 5 (15.6) |  |  |  |
| **Psychopathology [mean (SD)]** | | | | | |
| Positive and Negative Syndrome Scale - Positive | 20.7 (5.5) | 18.3 (7.0) | 54.830 | 1.597 | 0.116 |
| Positive and Negative Syndrome Scale - Negative | 14.6 (5.5) | 14.9 (5.5) | 62.032 | -0.237 | 0.814 |
| Positive and Negative Syndrome Scale - General | 35.0 (8.2) | 33.0 (6.0) | 71.438 | 1.216 | 0.228 |
| Onset Age of Psychotic Disorder | 23.0 (3.6) | 23.3 (4.1) | 58.594 | -0.262 | 0.794 |
| Years between first cannabis use initiation and attenuated psychotic symptoms – years [mean (SD)] | 6.9 (3.8) | 3.6 (4.4) | 33.676 | 2.911 | 0.006 |
| Years between initiation of heaviest cannabis use and attenuated psychotic symptoms – years [mean (SD)] | 2.3 (3.2) | 1.4 (2.8) | 55.378 | 1.090 | 0.280 |
| **Medication [mean (SD)]** | | | | | |
| Currently treated (%) | 28 (59.6) | 20 (62.5) | 1 | 0.040 | 0.841 |
| Chlorpromazine equivalent (cumulative lifetime) | 4276.4 (7196.8) | 4894.2 (8469.3) | 57.558 | -0.332 | 0.741 |

## ***sTable 10 Voxel based morphometry Analysis Results***

| **Brain region** | **MNI coordinates** | | | ***t*_max_** | ***z*_max_** | **Cluster size (voxels)** |
| --- | --- | --- | --- | --- | --- | --- |
|  | **x** | **y** | **z** |  |  |  |
| Cerebellum anterior lobe, Culmen, Vermis 4/5 | 0 | -50 | -2 | 3.11 | 3.02 | 54 |
| Cerebellum posterior lobe, Uvula | 28 | -80 | -36 | 2.93 | 2.86 | 37 |

#

# ***Supplementary - Figures***

**
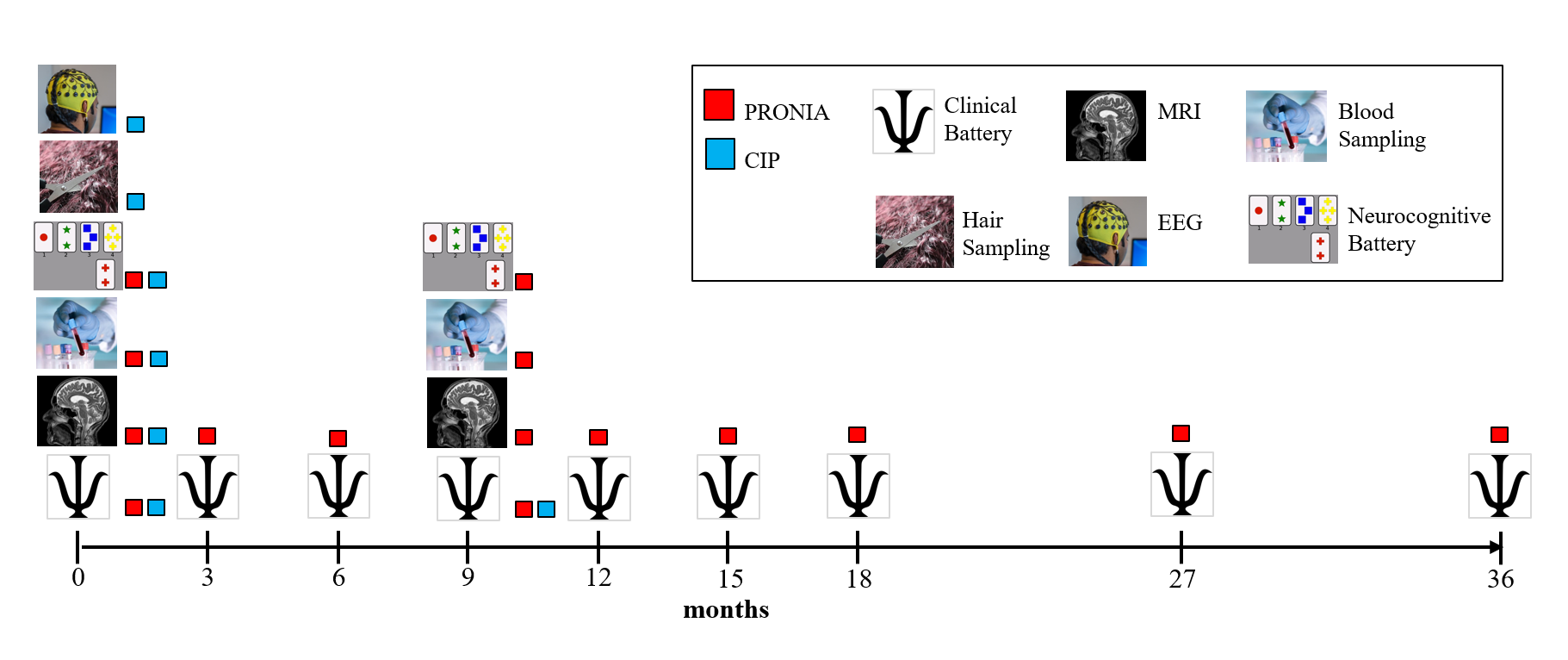
**

## *sFigure 1 Design of PRONIA and CIP studies (Figure adapted from* [*^12^*](https://paperpile.com/c/Vzk3fL/DLga)*) The colored boxes indicate the study containing each assessment.*

| **SUBSTANCE USE**  (dd-mm-yyyy) | **- -** | |
| --- | --- | --- |
| **Cannabis lifetime**  *(follow-up: since the last assessment)* | 0 = No 1 = Yes | Date of onset: Date of offset:  (ongoing: 66-66-6666) |
| **Cumulative number of months:** |  | |
| **Daily/weekly frequency of use (average) during the last 3 months**  *(follow-up: since the last visit)* | 1 = daily  2 = > 3 days a week 3 = <= 3 days a week 4 = less than weekly 5 = never |  |
| **Cannabis - cumulative frequency of use during the last 3 months**  (follow-up: since the last visit) | 1 = 1-5 times  2 = 6-10 times  3 = 11-15 times  4 = 16-20 times  5 = 21-30 times  6 = > 30 times  7 = not applicable |  |
| **Last consumption**  (dd-mm-yyyy) | **- -** | |
| **Other substances lifetime**  *(follow-up: since the last visit)* | 1 = hallucinogens  2 = cocaine  3 = amphetamine-type stimulants incl. MDMA  4 = inhalants  5 = opioids  6 = PCP or similar type  7 = other designer drugs  8 = sedative-yypnotic- anxiolytic  9 = none |  |
| **Other substances – daily/weekly frequency of use (average) during the last 3 months**  *(follow-up: since the last visit)* | 1 = daily  2 = > 3 days a week 3 = <= 3 days a week 4 = less than weekly 5 = never |  |
| **Other substances - cumulative frequency of use during the last 3 months (different drugs can be added)**  *(follow-up: since the last visit)* | 1 = 1-5 times  2 = 6-10 times  3 = 11-15 times  4 = 16-20 times  5 = 21-30 times  6 = > 30 times  7 = not applicable |  |
| **Last consumption**  (dd-mm-yyyy) | **- -** | |

## *sFigure 2 Substance Use Questionnaire*

**
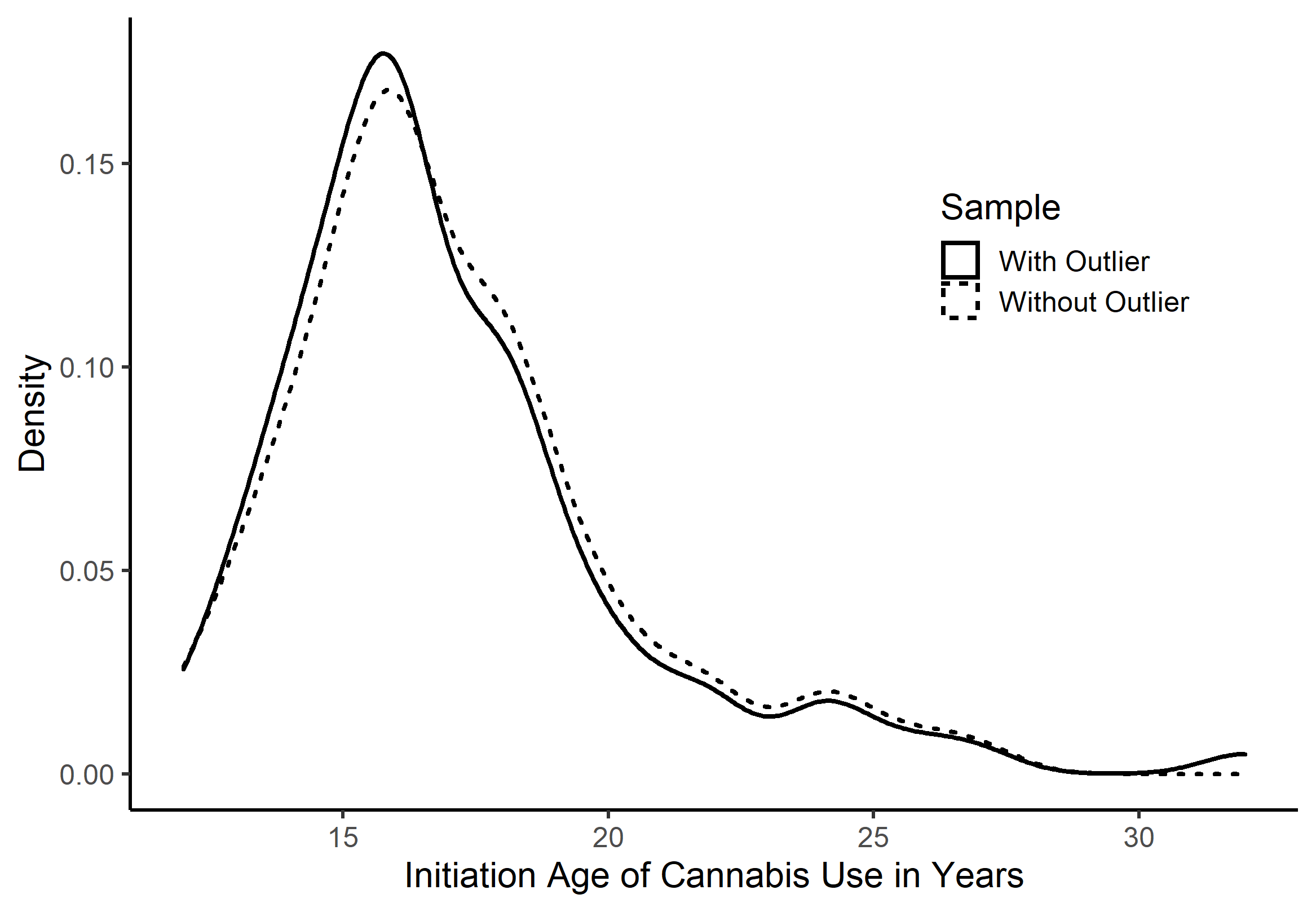
**

## *sFigure 3 Distribution of age of initiation. The distribution of age of cannabis use initiation is shown for the whole sample, with (n = 102) and without (n = 95) inclusion of significant outliers in initiation age and duration of heaviest use.*

**
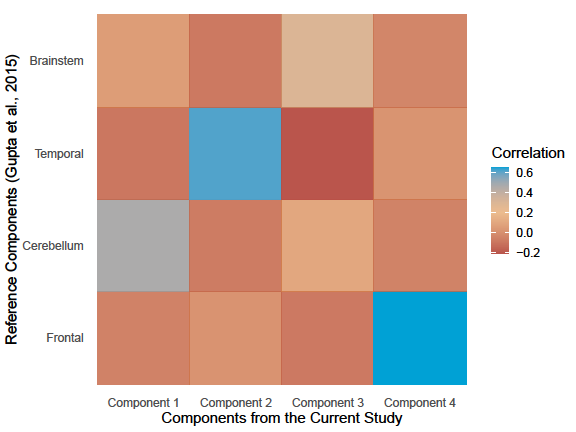
**

## *sFigure 4 Correlation between the components from the current study and the reference components - The heatmap represents the pairwise correlation between the components. Thereby, all non-zero voxels were correlated with each other.*

**
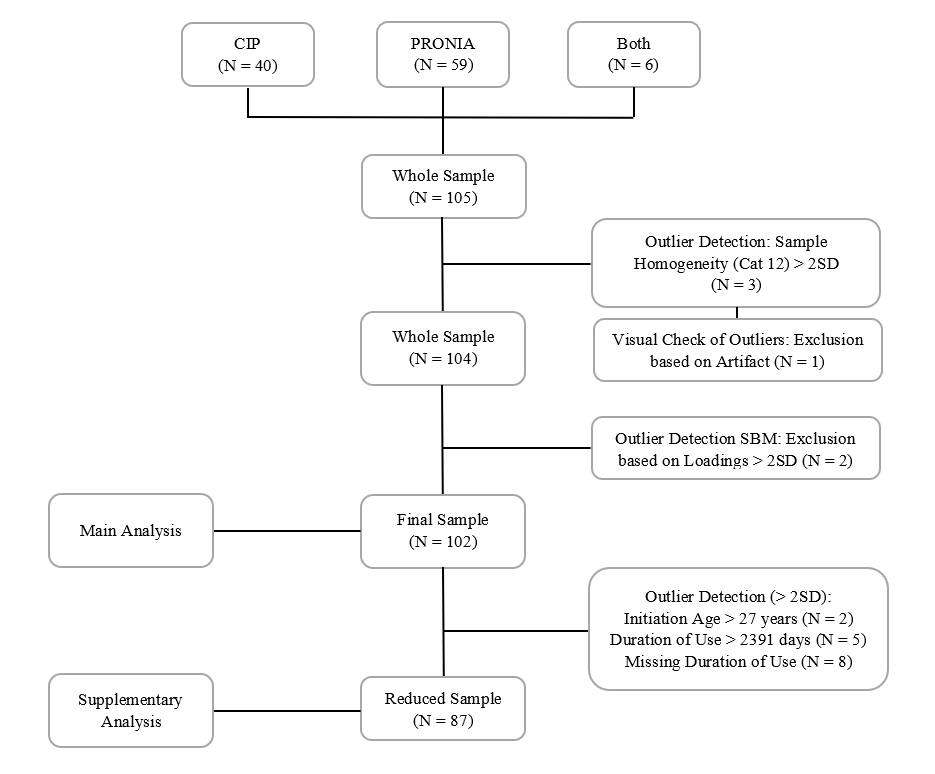
**

## *sFigure 5 Flow Diagram - Inclusion based on Outlier*

**
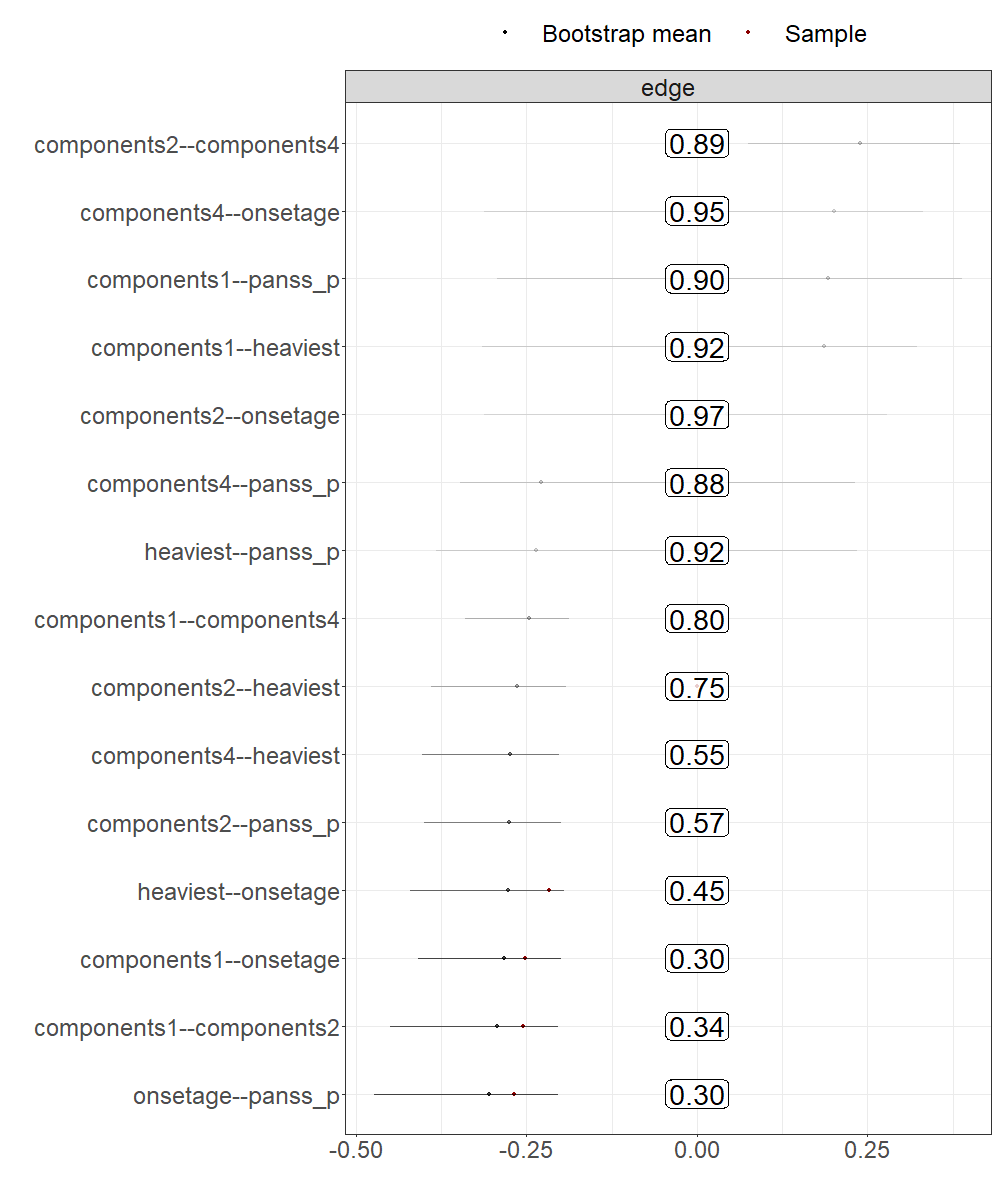
**

## *sFigure 6 Results of bootstrapping the network 1000 times. Numbers in squares represent how often an edge was set to zero, i.e., not included in the network. This output shows that the edges retained in our final model were also included in the majority of bootstrapped networks.*

**
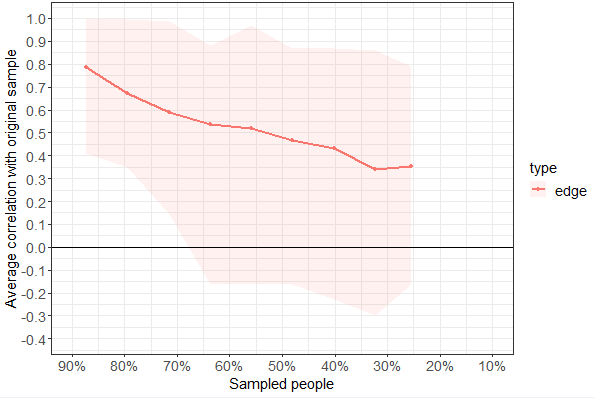
**

## *sFigure 7 Stability of edge weights testing by case dropping subset bootstrapping for the six-item network. The x-axis depicts the percentage of cases of the original sample used at each step. The y-axis depicts the average of correlations between the edge weights from the original network with the edge weights from the networks that emerged after dropping x-percentage of cases. The maximum proportion of observations that could be dropped while confidently (95%) retaining results of high correlation (r > .7) with centrality estimates in the original sample was 0 %, indicating low stability.*

#


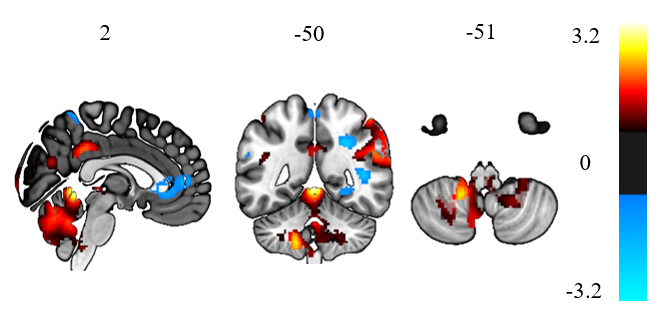


## *sFigure 8 Results of the VBM analysis – Correlation between age of cannabis initiation and GM volume; voxels threshold at |Z| > 1.5 are shown.*

# ***Supplementary – References***

[1] Spitzer MB, Gibbon, Robert, M., Gibbons W, Janet BW, Gibbon, Miriam, R. L., Williams, J. B. W. Structured Clinical Interview for DSM-IV-TR Axis I Disorders, Research Version, Non-Patient Edition.(SCID-I/NP). 2002.

[2] Chen J, Liu J, Calhoun VD, Arias-Vasquez A, Zwiers MP, Gupta CN, et al. Exploration of scanning effects in multi-site structural MRI studies. J Neurosci Methods 2014;230:37–50.

[3] Gupta CN, Calhoun VD, Rachakonda S, Chen J, Patel V, Liu J, et al. Patterns of Gray Matter Abnormalities in Schizophrenia Based on an International Mega-analysis. Schizophr Bull 2015;41:1133–42.

[4] Koutsouleris N, Kambeitz-Ilankovic L, Rosen M, Ruef A, Dwyer, Dominic, B., Chisholm K, et al. Individualized Prediction of Functional Outcomes in Mental Healthcare: A Multi-modal, Multi-Site Machine Learning Analysis in the Clinical High-Risk State for Psychosis and Recent-Onset Depression: Supplementary Methods and Results. JAMA Psychiatry 2018;75:1–20.

[5] Du Y, Lin D, Yu Q, Sui J, Chen J, Rachakonda S, et al. Comparison of IVA and GIG-ICA in Brain Functional Network Estimation Using fMRI Data. Front Neurosci 2017;11:267.

[6] Gaser C. CAT-a computational anatomy toolbox for the analysis of structural MRI data.: Gaser, C., & Dahnke, R. (2016). HBM, 2016, 336-348. HBM 2016:336–48.

[7] Gaser C, Kurth F. Manual computational anatomy toolbox-CAT12. *Structural Brain Mapping Group at the Departments of Psychiatry and Neurology, University of Jena*. 2017.

[8] Grubbs FE. Procedures for Detecting Outlying Observations in Samples. technometrics 1969;11 (1):1–21.

[9] Genovese CR, Lazar NA, Nichols T. Thresholding of statistical maps in functional neuroimaging using the false discovery rate. Neuroimage 2002;15:870–8.

[10] Betz LT, Penzel N, Rosen M, Kambeitz J. Relationships between childhood trauma and perceived stress in the general population: a network perspective. Psychol. Med. 2020:1–11.

[11] Epskamp S, Borsboom D, Fried EI. Estimating psychological networks and their accuracy: A tutorial paper. Behav Res Methods 2018;50:195–212.

[12] Stanfield AC, McIntosh AM, Spencer MD, Philip R, Gaur S, Lawrie SM. Towards a neuroanatomy of autism: A systematic review and meta-analysis of structural magnetic resonance imaging studies. Eur Psychiatry 2008;23:289–99.

[13] Valera EM, Faraone SV, Murray KE, Seidman LJ. Meta-analysis of structural imaging findings in attention-deficit/hyperactivity disorder. Biol Psychiatry 2007;61:1361–9.

[14] Tiemeier H, Lenroot RK, Greenstein DK, Tran L, Pierson R, Giedd JN. Cerebellum development during childhood and adolescence: A longitudinal morphometric MRI study. Neuroimage 2010;49:63–70.

[15] Hamilton I, Galdas P, Essex H. Cannabis psychosis, gender matters. Advances in Dual Diagnosis 2015;8:153–62.

[16] Duperrouzel JC, Granja K, Pacheco-Colón I, Gonzalez R. Adverse Effects of Cannabis Use on Neurocognitive Functioning: A Systematic Review of Meta- Analytic Studies. J Dual Diagn 2019:1–15.

[17] Gorey C, Kuhns L, Smaragdi E, Kroon E, Cousijn J. Age-related differences in the impact of cannabis use on the brain and cognition: A systematic review. Eur Arch Psychiatry Clin Neurosci 2019;269:37–58.

[18] Schmidt M. Rey auditory verbal learning test: A handbook. Los Angeles: CA: Western Psychological Services; 1996.

[19] Benedict RHB, Schretlen D, Groninger L, Brandt J. Hopkins Verbal Learning Test – Revised: Normative Data and Analysis of Inter-Form and Test-Retest Reliability. The Clinical Neuropsychologist 2010;12:43–55.

[20] Nuechterlein KH, Green MF, Kern RS, Baade LE, Barch DM, Cohen JD, et al. The MATRICS Consensus Cognitive Battery, Part 1: Test Selection, Reliability, and Validity. Am J Psychiatry 2008:203–13.

[21] Kern RS, Nuechterlein KH, Green MF, Baade LE, Fenton WS, Gold JM, et al. The MATRICS Consensus Cognitive Battery, Part 2: Co-Norming and Standardization. Am J Psychiatry 2008:214–20.

[22] Haas SS, Antonucci LA, Wenzel J, Ruef A, Biagianti B, Paolini M, et al. A multivariate neuromonitoring approach to neuroplasticity-based computerized cognitive training in recent onset psychosis. Neuropsychopharmacology 2020.

[23] Schultze-Lutter F, Addington J, Ruhrmann S, Klosterkötter J. Schizophrenia Proneness Instrument: Adult version (SPI-A). Rome: Giovanni Fioriti; 2007.

[24] McGlashan TH, Miller TJ, Woods SW, Hoffman RE, Davidson L. Instrument for the assessment of prodromal symptoms and states. Early Intervention in Psychotic Disorders 2001:135–49.

[25] Alison R. Yung, Hok Pan Yuen, Patrick D. Mcgorry, Lisa J. Phillips, Daniel Kelly, Margaret Dell'olio, et al. Mapping the Onset of Psychosis: The Comprehensive Assessment of At-Risk Mental States.

[26] Startup M, Jackson MC, Bendix S. The concurrent validity of the Global Assessment of Functioning (GAF). The British Journal of Psychological Society 2002:417–22.

[27] Alvarez E, Garcia-Ribera C, Torrens M, Udina C, Guillamat R, Casas M. Premorbid Adjustment Scale as a Prognostic Predictor for Schizophrenia. The British Journal of Psychiatry;1987.

[28] Kay SR, Fiszbein A, Opler LA. The Positive and Negative Syndrome Scale (PANSS) for Schizophrenia. Schizophr Bull 1987;13:261–76.

[29] Andreasen NC. The Scale for the Assessment of Negative Symptoms (SANS): Conceptual and Theoretical Foundations. British Journal of Psychiatry;1989:49–52.

[30] Llorca P-M, Lançon C, Lancrenon S, Bayle F-J, Caci H, Rouillon F, et al. The "Functional Remission of General Schizophrenia" (FROGS) scale: Development and validation of a new questionnaire. Schizophr Res 2009;113:218–25.

[31] Cornblatt BA, Auther AM, Niendam T, Smith CW, Zinberg J, Bearden CE, et al. Preliminary findings for two new measures of social and role functioning in the prodromal phase of schizophrenia. Schizophr Bull 2007;33:688–702.

[32] Zimet GD, Powell SS, Farley GK, Werkman S, Berkoff KA. Psychometric characteristics of the Multidimensional Scale of Perceived Social Support. J Pers Assess 1990;55:610–7.

[33] Friborg O, Hjemdal O, Rosenvinge JH, Martinussen M. A new rating scale for adult resilience: what are the central protective resources behind healthy adjustment? Int J Methods Psychiatr Res 2003;12:65–76.

[34] Endler NS, Parker JD. Multidimensional assessment of coping: A critical evaluation. Journal of Personality and Social Psychology 1990;58:844–54.

[35] Connor KM, Davidson JRT, Churchill LE, Sherwood A, Foa E, Weisler RH. Psychometric properties of the Social Phobia Inventory (SPIN): New self-rating scale. British Journal of Psychiatry 2000;176:379–86.

[36] Beck AT, Steer RA, Brown GK. Beck depression inventory-II. San Antonio, 78(2), 490-8. San Antonio 1996;78:490–8.

[37] WHO. WHOQOL-BREF: Introduction, Administration, Scoring and Generic Version of the Assessment. WHO Division of Mental Health;1996.

[38] Veale JF. Edinburgh Handedness Inventory - Short Form: A revised version based on confirmatory factor analysis. Laterality 2014;19:164–77.

[39] Cole JD, Kazarian SS. The level of expressed emotion scale: A new measure of expressed emotion. Journal of Clinical Psychology 1988;44:392–7.

[40] Klein MH, Benjamin LS, Rosenfeld R, Treece C, Husted J, Greist J. The Wisconsin Personality Disorders Inventory: Development, Reliability and Validity. Journal of Personality Disorders 1993;7(4):285–303.

[41] David R. Williams, Yan Yu, James S. Jackson, and Norman B. Anderson. Racial Differences in Physical and Mental Health.

[42] Haidl TK, Schneider N, Dickmann K, Ruhrmann S, Kaiser N, Rosen M, et al. Validation of the Bullying Scale for Adults - Results of the PRONIA-study. J Psychiatr Res 2020;129:88–97.

[43] Bernstein DP, Fink L, Handelsman L, Foote J. Childhood Trauma Questionnaire: Assessment of family violence: A handbook for researchers and practitioners. APA PsycTests; 1998.

[44] Costa PT, McCrae RR. The NEO Inventories. Routledge/Taylor & Francis Group; 2008.

[45] Cornblatt BA, Risch NJ, Faris G, Friedman D, Erlenmeyer-Kimling L. The Continuous Performance Test, Identical Paris Version (CPT-IP): I. New Findings About Sustained Attention in Normal Families. Psychiatry Res 1988;26:223–38.

[46] Nowicki S, Duke MP. Individual differences in the nonverbal communication of affect: The diagnostic analysis of nonverbal accuracy scale. J Nonverbal Behav 1994;18:9–35.

[47] Rey A. L'examen Psychologique Dans les cas D'encephalopathie Traumatique (Les Problems),. Archives de Psychologie 1941:215–85.

[48] Roiser JP, Stephan KE, den Ouden HEM, Friston KJ, Joyce EM. Adaptive and aberrant reward prediction signals in the human brain. Neuroimage 2010;50:657–64.

[49] Petrides M, Milner B. Deficits on subject-ordered tasks after frontal- and temporal-lobe lesions in man. Neuropsychologia 1982;20:249–62.

[50] Reitan RM. Trail Making Test: Manual for administration and scoring; 1992.

[51] Wechsler D. WAIS-3., WMS-3: Wechsler adult intelligence scale, Wechsler memory scale: Technical manual; 1997.
